# HPV Genotyping and Site of Viral Integration in Cervical Cancers in Indian Women

**DOI:** 10.1371/journal.pone.0041012

**Published:** 2012-07-16

**Authors:** Poulami Das, Asha Thomas, Umesh Mahantshetty, Shyam K. Shrivastava, Kedar Deodhar, Rita Mulherkar

**Affiliations:** 1 Mulherkar Lab, Advanced Centre for Treatment, Research and Education in Cancer (ACTREC), Tata Memorial Centre, Navi Mumbai, India; 2 Department of Radiation Oncology, Tata Memorial Hospital, Tata Memorial Centre, Mumbai, India; 3 Department of Pathology, Tata Memorial Hospital, Tata Memorial Centre, Mumbai, India; Karolinska Institutet, Sweden

## Abstract

Persistent HPV infection plays a major role in cervical cancer. This study was undertaken to identify HPV types in a cohort of Indian women with locally advanced cervical cancer as well as to determine the physical state and/or site of viral integration in the host genome. Pretreatment biopsies (n = 270) from patients were screened for HPV infection by a high throughput HPV genotyping assay based on luminex xMAP technology as well as MY09/11 PCR and SPF1/2 PCR. Overall HPV positivity was observed to be 95%, with HPV16 being most common (63%) followed by infection with HPV18. Integration status of the virus was identified using Amplification of Papillomavirus Oncogene Transcripts (APOT) assay in a subset of samples positive for HPV16 and/or HPV18 (n = 86) and with an adequate follow-up. The data was correlated with clinical outcome of the patients. Integration of the viral genome was observed in 79% of the cases and a preference for integration into the chromosomal loci 1p, 3q, 6q, 11q, 13q and 20q was seen. Clinical data revealed that the physical state of the virus (integrated or episomal) could be an important prognostic marker for cervical cancer.

## Introduction

Cervical cancer is the third most common cancer among women worldwide and the most common cancer found in Indian women. HPV infection has been shown to play a critical, though not sufficient, role in the etiology of cervical cancers. Till date more than 200 HPV types have been reported, of which HPV16 is most common, followed generally by HPV18, HPV45, HPV31 and HPV33 [1], [2]. Most of the high risk HPV (HR-HPV) infections (90%) regress spontaneously and only in about 10% cases the infection persists and progresses to high-grade cervical intraepithelial neoplasia. This generally occurs through integration of the HPV genome into the host chromosome with associated loss or disruption of E2 [3]. According to available reports, viral E2 gene has the ability to repress viral E6 and E7 oncogenes in cells harbouring integrated HPV DNA [4]. Therefore integration of the virus with loss of transcriptional control by E2 results in overexpression of E6 and E7 leading to immortalization and transformation of cells [5]. In most of the cases integration of HR-HPV genome gives rise to fusion transcripts comprising of viral oncogenes E6, E7 and adjacent cellular sequences [6], [7], [8], [9]. In vitro studies have demonstrated that the viral-cellular fusion transcripts are more stable and impart the cells with a selective growth advantage as compared to the episomal counterparts [10], [11].

Studies report that the integration event is random involving almost all the chromosomes, and accordingly several virus-host integration sites have been mapped till date [12]. However, there are certain hotspots e.g., fragile sites, translocation break points and transcriptionally active regions [3], [13], [14], [15] which are preferred by the virus for its integration into the human genome. On integration within or near a gene, the virus can bring about a change in its expression which may eventually lead to alterations in cellular growth and proliferation. Also, viral integration can render both viral coding genes as well as the cellular genes susceptible to epigenetic changes which could regulate their expression. Hence, the integration of HPV into the human genome is considered an important event in cervical carcinogenesis.

The aim of this study was HPV genotyping and identification of site of integration of two HR-HPV types (16 and 18), along with evaluation of the prognostic value of integration site, in locally advanced cervical cancers in Indian women.

## Materials and Methods

### Clinical Samples

Pretreatment cervical tumor biopsies, predominantly from FIGO stage IIIB, were obtained from patients (median age, 50 years; age range, 33–80 years) undergoing radiotherapy alone or concomitant chemo-radiation at the Radiation Oncology Department, Tata Memorial Hospital, Mumbai, after obtaining IRB approval. A generic consent for basic research was obtained prior to obtaining the biopsies. However, for the current study a consent waiver was obtained from the Hospital Ethics Committee. The biopsies were obtained from histologically proven, primary cervical tumor, before the start of radical radiation therapy and were coded for de-identification by the physician prior to testing. The samples were collected in liquid nitrogen and stored at −80°C until further use. All the samples were assigned a laboratory code to maintain confidentiality.

### Processing of tumor samples

Frozen tissues were cryocut for extraction of DNA and RNA. For DNA extraction, five 30 µm sections were collected in STE buffer (0.1 M NaCl, 0.05 M Tris pH 7.5, 1 mM EDTA, 1% SDS) containing 10 mg/ml Proteinase K (USB, Cleveland, OH, US). DNA was isolated by standard phenol-chloroform method. For isolation of total RNA, RNeasy Mini Kit (Qiagen, Hilden, Germany) was used. Ten 30 µm sections were collected in RLT buffer containing guanidine thiocyanate provided in the kit and processed following manufacturer's instructions. DNAse treatment of the RNA samples was carried out using DNA free kit (Ambion, Austin, TX, US).

### HPV genotyping by high throughput luminex array

Genotyping of 24 HPV types which included 15 high-risk types (16, 18, 31, 33, 35, 39, 45, 51,52, 56, 58, 59, 68, 73, and 82), 3 putative high-risk types (26, 53, and 66), and 6 low-risk types (6, 11, 42, 43, 44, and 70) [1], [2] was carried out using multiplex HPV genotyping array (Multimetrix GmbH, Heidelberg, Germany) based on luminex xMAP technology. As per the manufacturer's instructions, PCR was carried out using sets of biotinylated broad range primers in a total volume of 50 µL containing 3.5 mM MgCl_2_, 200 µM dNTPs, 0.75 unit of Taq DNA polymerase and 1µl primer mix. The amplification steps included an initial DNA denaturation at 94°C for 5 min, followed by 40 cycles of denaturation for 20 s at 94°C, annealing for 30 s at 38°C, and extension for 1 min 20 s at 71°C, before a final extension for 4 min at 71°C. PCR positive samples were then subjected to the luminex run. Ten microlitres of the PCR product was mixed with the luminex bead mix containing distinct bead populations coupled to 24 HPV types. After thermal denaturation, the target sequences were hybridized to bead-bound probes. The hybridized PCR products were labeled by binding to R-phycoerythrin conjugated streptavidin. The read-out was obtained in the luminex bioanalyzer (Luminex Corporation, Austin, TX, USA). HPV types were discerned according to the unique bead signature, whereas the presence of PCR products was determined by phycoerythrin fluorescence. An analytical sensitivity cut-off was calculated based on the negative control which was deducted from each of the read-out.

### HPV genotyping by PCR using MY09/11 and SPF1/2 primers

Since the amount of DNA available for the study was limiting, β-actin PCR was done only in those samples which failed to show amplification by GP5^+^/6^+^ primers (n = 92). These were further screened for HPV by PCR using MY09/11 L1 primers (Table S1) and SPF1/2 primers [16]. PCR was carried out in a reaction volume of 25 μl containing, 1.5 mM MgCl_2_, 10μM of each primer, 200 μM dNTPs and 0.75 unit of Taq DNA polymerase. The samples which tested positive for HPV either by MY09/11 or SPF1/2 or both were further genotyped for the two most common HR-HPV types- HPV16 and 18 using HPV16/18 specific primers (Table S1). Since the amount of DNA was limiting, SPF 1/2 PCR could not be carried out in 4 of the 92 samples.

### Association of HPV16, HPV18 and HPV16/18 infection with clinical outcome

The genotyping data for the two HR-HPV types, HPV16, HPV18 and HPV16/18 together, where adequate follow-up data was available was compared with the clinical outcome of the patients. Kaplan-Meier analysis (SPSS 15.0) was done to determine association between infection with these HR-HPV types and recurrence of disease. Disease free survival was considered from start of radiation therapy to the time when recurrence occurred or till last follow-up. Statistical significance was evaluated using the log-rank test (SPSS 15.0).

### Identification of integration site by APOT assay

Samples (n = 86) positive for HPV16, HPV18 or both and with an adequate clinical follow-up (minimum 1yr or till occurrence of first event, whichever was earlier) were taken to study viral integration using Amplification of Papillomavirus Oncogene Transcripts (APOT) assay, as described by Klaes *et al* [6]. Briefly, total RNA (0.5–1 µg) was reverse transcribed using oligo(dT)17-primer coupled to a linker sequence [(dT)17–p3] [17] and 50 units of Superscript II reverse transcriptase (Invitrogen, Carlsbad, CA, USA) for 1 h at 42°C. PCR with β-actin primers was carried out to check the integrity of the cDNA. First strand cDNA was amplified using HPV E7–specific primer (p1–16 specific for HPV16 and p1–18 specific for HPV18) as forward primers and linker p3 as the reverse primer (Table S1). The PCR amplification was carried out in a reaction volume of 50 µL containing 2.5 mM MgCl_2_, 200 μM dNTPs, 25 µM of each primer and 1 unit of Taq DNA polymerase. The reaction comprised of an initial denaturation step at 94°C for 2 min, followed by 35 cycles of denaturation at 94°C for 30 s, annealing at 58°C for 30 s, and extension at 72°C for 4 min. This was followed by a final extension at 72°C for 20 min. Next, 7 µl of the PCR product was used as template for nested PCR using forward primers p2–16 specific for HPV16 or p2–18 specific for HPV18 and (dT)17–p3 as reverse primer (Table S1). The PCR conditions were same as that of first PCR except that the annealing temperature was 66°C.

### Cloning of fusion transcripts

Amplicons other than the major episomal transcripts (∼1050 bp for HPV16 and ∼1000 bp for HPV18) were suspected to be derived from the integrated HPV genomes. These were excised from the gel and DNA isolated using GFX PCR DNA and Gel Band Purification Kit (GE Healthcare, Buckinghamshire, UK). The isolated DNA was either sequenced directly or following cloning into pTZ57R/T vector using the InsTA PCR Cloning Kit (Fermentas, Lithuania, EU), on DNA sequencer (3100 Avant Genetic analyzer, Applied Biosystems, Foster City, CA, USA). The chromosomal integration loci were determined using National Centre for Biotechnology Information (BLAST) and the University of California, Santa Cruz (UCSC) hg19 (Feb. 2009) (BLAT) human genome assemblies. Further, the integration sites were checked for the presence of fragile sites and any genes of known identity by using NCBI fragile site map viewer and the UCSC Blat tool respectively.

### Association of viral integration with clinical outcome

The data obtained was compared with the clinical outcome of the patients. Kaplan-Meier analysis (SPSS 15.0) was done to determine the association of the viral state (episomal/integrated) with recurrence of the disease. Disease free survival was considered from start of radiation therapy to the time when recurrence occurred or till last follow-up (median follow-up for 86 cases was 44 months). Statistical significance was evaluated using the log-rank test (SPSS 15.0).

## Results

### HPV genotyping by high throughput luminex array

**Figure 1:**
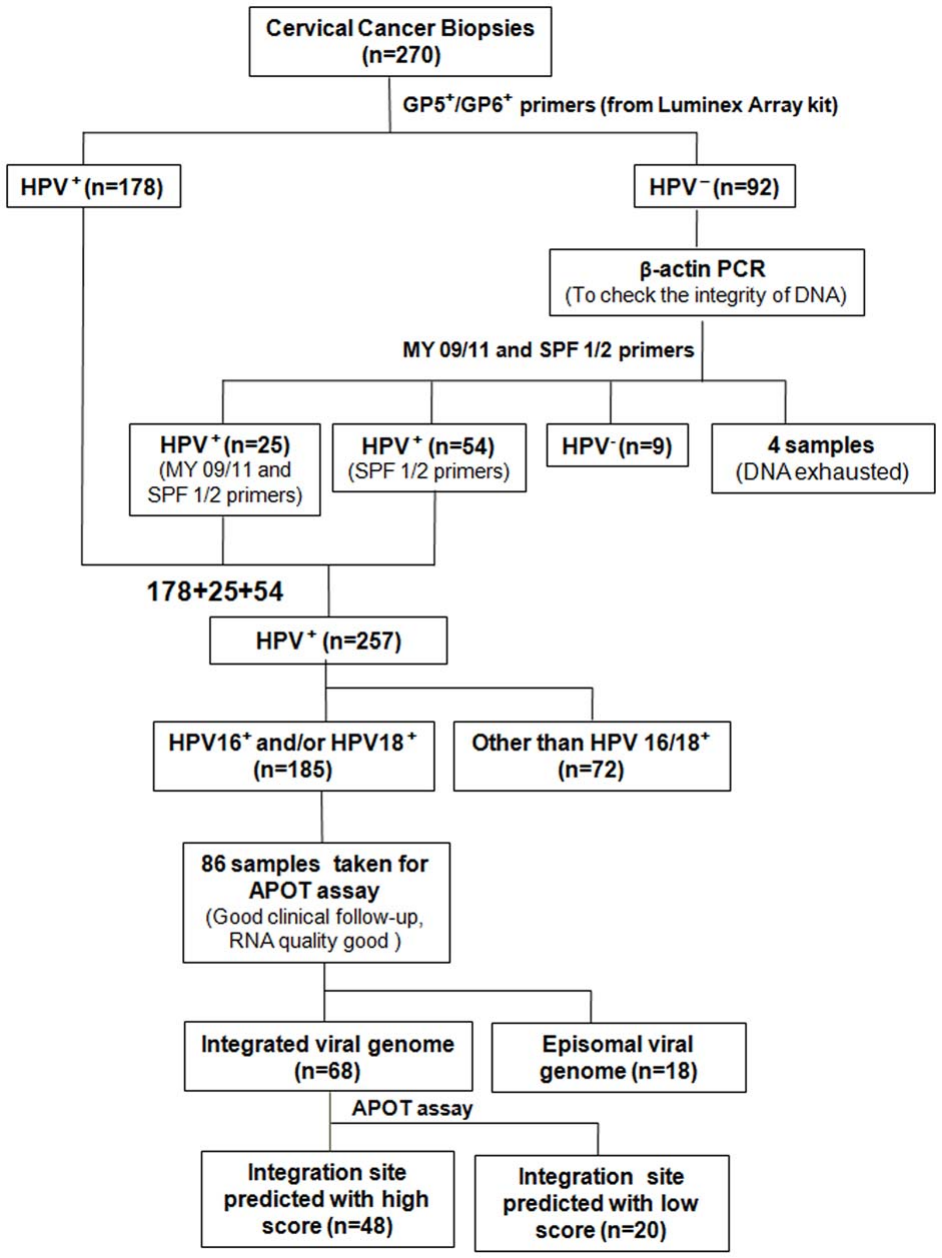
Flowchart depicting summary of the study. Genotyping was carried out on 270 advanced stage cervical cancer samples by high-throughput, GP5^+^/6^+^ primers based luminex array; consensus MY09/11 and SPF1/2 primers. HPV positivity was 95% (257/270). APOT assay was done on 86 HPV16^+^ and/or HPV18^+^ samples, with good clinical follow-up and good quality RNA. In 18 samples, only episomal form of HPV was identified, rest 68 hinted toward possible integration. Site of integration could be predicted with high score by BLAST and/or BLAT in 48 samples.

**Figure 2:**
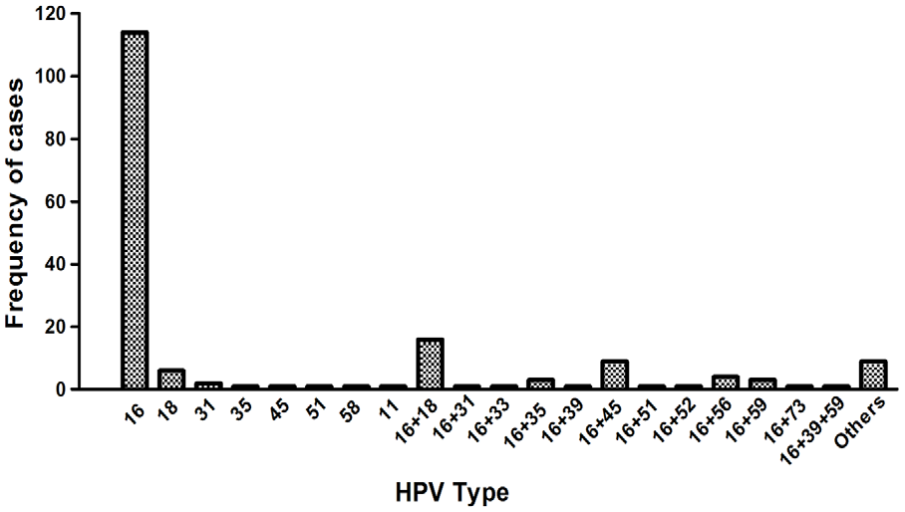
Frequency of 24 HPV types as detected by Luminex array. The graph depicts frequency of 24 HPV types in 178 cervical cancer biopsy samples which were found to be positive GP5^+^/6^+^ primers. HPV16 infection predominated in the samples. Each bar represents different HPV types.

Although there are a few reports on different high risk HPVs in Indian women, here we report 15 high risk HPVs, 3 intermediate risk and 6 low risk HPV types using the high throughput luminex array. The primers provided in the luminex array kit for HPV genotyping were biotinylated, broad range GP5^+^/GP6^+^ primers. Using this primer set for PCR, we obtained 178 out of 270 samples positive for HPV (Figure 1). The HPV positive samples were further subjected to hybridization to bead-bound probes by luminex array as described earlier. One hundred sixty nine samples were found to hybridize to the different HPV probes whereas 9 samples were negative. These 9 samples could have HPV infection not included in the 24 types detected by the kit. Out of 169 HPV positive samples, 168 samples were positive for one or more HR-HPV types indicating a high association of cervical cancer with HR-HPV infection. Among these, HPV16 and/or HPV18 infection were most common – 114 samples being positive for HPV16 alone, 6 samples for HPV18 alone and 16 samples for both HPV16 and HPV18 (Figure 2).

### HPV genotyping by PCR using MY09/11 and SPF1/2 primers

In order to estimate the true HPV positivity in the 270 cases, the 92 cervical cancer biopsies negative for HPV by luminex array, were first subjected to PCR using β-actin primers to check the quality of DNA. All were found to be positive for β-actin. Next they were subjected to PCR using MY09/11 and SPF1/2 primers. Twenty five out of 92 samples were found to be positive for HPV by MY09/11 PCR. Since the amount of DNA was limiting, SPF 1/2 PCR could not be carried out in 4 of the 92 samples. Screening with SPF1/2 primers revealed 79 samples to be HPV positive. These 79 samples also included the 25 samples that tested HPV positive by MY09/11 PCR. The HPV positivity was therefore calculated taking into account the results from luminex array and SPF1/2 PCR. The overall HPV positivity in this cohort was found to be 95% (257/270). Further genotyping of the 79 samples, using HPV 16/18 specific primers, showed 49 samples to be positive for HPV16. None of these 79 samples were positive for HPV18. Therefore, the prevalence of HPV16 and/or HPV18 in this cohort was 69% (185/270) (Figure 1).

### Association of HPV16, 18 and dual infection with clinical outcome

Kaplan-Meier survival analysis data for 125 patients with HPV type16, 18 and dual infection and with adequate clinical follow-up (median follow-up for 125 cases was 54 months), revealed that there was no significant difference between infection with these two HR-HPV types in terms of disease outcome (Figure S1).

### Physical state of virus and clinical outcome

**Figure 3:**
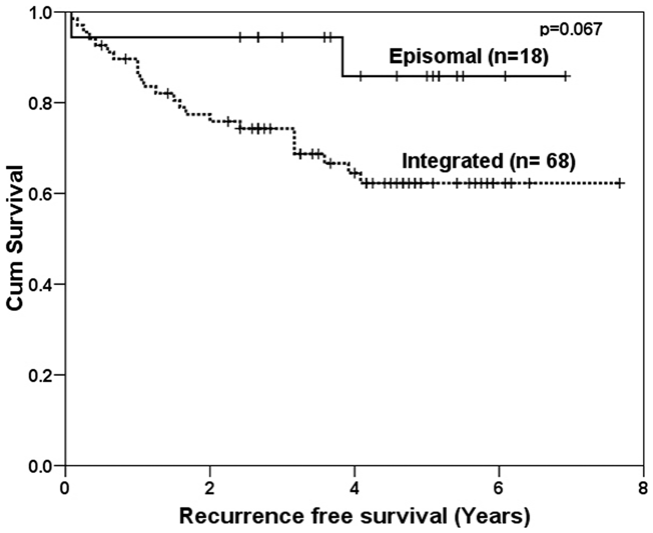
Kaplan-Meier analysis for episomal vs. integrated viral genome. Kaplan-Meier survival curve for patients with episomal form of virus (n = 18) vs. integrated form (n = 68) is depicted. Most of the patients with episomal form (16 out of 18) had a disease free survival as compared to patients with integrated form, indicating a good clinical outcome, although with a borderline significance (p = 0.067).

Out of 125 HPV16, HPV18 or dual HPV positive samples, a sub-set of 86 samples with good quality RNA, were taken to study the physical state and/or site of viral integration by APOT assay. In most of the cases, the viral genome was found to be integrated (n = 68), whereas in 21% (n = 18) only episomal transcripts could be identified. In 12 cases with integrated viral genome, episomal form of HPV was also detected (Figure 1). The physical state of the virus (episomal/ integrated) was associated with the disease outcome. Survival data revealed that 16 out of 18 patients with only episomal form of HPV (16 and/or 18), had disease free survival as compared to those with integrated form of the virus, indicating a good clinical outcome (p = 0.067, representing a borderline significance) (Figure 3). The clinical outcome of all the patients where the viral integration was studied is shown in Table S2.

### Identification of viral integration site

**Figure 4:**
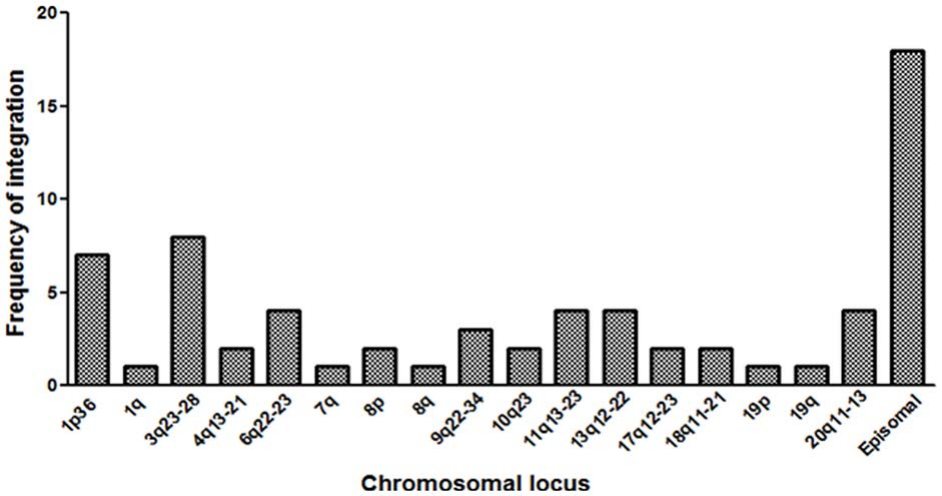
Frequency of HPV integration into different chromosomal loci. Site of integration as determined by APOT assay in 48 cases positive for HPV16, HPV18 or both and with high prediction score using BLAST/BLAT. Integration event was found to be more common in 1p and 3q chromosomal loci. Each bar represents different chromosomal locus.

In order to understand whether the integration event is random or there is some preference for certain sites within the chromosomes, the sequencing data for 68 cases derived from APOT assay were investigated by Blast and/or Blat. The site of integration could be predicted with a high score in 48 cases (Table S3a), for the remaining 20 cases the score was low (Table S3b). Only those cases where the integration site was predicted with high score (n = 48) were analyzed further for different features associated with the same. The sites of integration were found to be distributed throughout the genome. However, integration was more frequent at the chromosomal loci 1p (n = 7), 3q (n = 8), 13q (n = 4), 6q (n = 4), 11q (n = 4) and 20q (n = 4) (Figure 4). Only one sample showed HPV integration at two chromosomal loci simultaneously. Some of the recurrent integration sites were also checked at the genomic level by carrying out genomic DNA PCR with HPV E7 primers as the forward primer and primers specific to a given chromosomal region as the reverse one (Figure S2).

### Features associated with HPV integration

Using NCBI Fragile site Map Viewer it was observed that 60% of integrations (29/48) were located in or close to a common or rare fragile site. The rest of the integration sites were not associated with any fragile sites (Table 1). Using the UCSC Blat tool 58% of the sequences (28/48) were observed to be either within or nearby protein coding genes. These genes belonged to various categories ranging from oncogenes, transcription factors, and tumor suppressor genes (Table 1).

**Table 1:** Summary of HPV Integration Sites.

Sample ID	Histology	HPV type	Integration locus	Gene	Fragile site involved
CT 712	SCC	16	1p36.12	ALPQTL2	FRA1A(1p36) com
CT 700	SCC	16	1p36.23	-	FRA1A(1p36) com
CT 702	SCC	16	1p36.23	-	FRA1A(1p36) com
CT 709	SCC	16	1p36.23	-	FRA1A(1p36) com
CT 1138	SCC	16	1p36.33	-	FRA1A(1p36) com
CT 809	SCC	16	1p36.33	-	FRA1A(1p36) com
CT 866	SCC	16	1p36.33	-	FRA1A(1p36) com
CT 864	SCC	16	1q42.13	ABCB10	FRA1H(1q42) com
CT 755	SCC	16	3q23	SLC25A36	-
CT 785	SCC	16	3q23	SLC25A36	-
			20q11.21	COX4I2	-
		18	-	-	-
CT 1210	SCC	16	3q26.2 and Episomal	MECOM	-
CT 723	SCC	16	3q26.31	-	FRA3C(3q27) com
CT 706	SCC	16	3q26.33	SOX2	FRA3C(3q27) com
CT 892	SCC	16	3q26.33	-	FRA3C(3q27) com
CT 711	SCC	16	3q28 and Episomal	LEPREL1	FRA3C(3q27) com
CT 739	SCC	16	3q28	TP63	FRA3C(3q27) com
CT 999	SCC	16	4q13.3	IL8(∼3kb)	-
CT 1114	SCC	16	4q21.22	HNRPDL	-
CT 893	SCC	16	6q22.31	-	-
CT 1117	AC	16	6q22.31	-	-
		18	-	-	-
CT 1122	SCC	16	6q23.3	PDE7B	-
CT 846	SCC	16	6q23.3	MAP3K	-
CT 1019	SCC	16	7q11.21	TYW1	FRA7J(7q11) com
CT 796	SCC	16	8p11.21	-	-
CT 915	SCC	16	8p11.21	-	-
		18	-	-	-

Sample ID	Histology	HPV type	Integration locus	Gene	Fragile site involved
CT 1160	SCC	16	8q24.21	MYC/PVT	Flanked by FRA8E(8q24.1) rare; FRA8C(8q24.1) com; FRA8D(8q24.3) com
CT 714	SCC	16	9q22.32	C9orf3	FRA9D(9q22.1) com
CT 1162	SCC	16	9q22.32 and Episomal	C9orf3	FRA9D(9q22.1) com
		18	-	-	-
CT 753	SCC	16	9q34.11	SLC25A25	-
CT 821	SCC	18	10q23.33	-	FRA10A(10q23.3) rare
CT 859	SCC	16	10q23.31	PTEN	FRA10A(10q23.3) rare
CT 1169	SCC	16	11q13.1 and Episomal	DRAP1	FRA11H(11q13) com
CT 1183	SCC	16	11q13.1	DRAP1	FRA11H(11q13) com
CT 836	SCC	16	11q14.3	-	FRA11F(11q14.2) com
CT 871	AC	18	11q23.3	ARCN1	FRA11G(11q23.3) com
CT 1094	SCC	16	13q	-	-
CT 927	SCC	16	13q12.3	POMP	-
CT 896	SCC	16	13q22.1	-	Flanked by FRA13B(13q21) com
CT 976	SCC	16	13q22.1	KLF5	Flanked by FRA13B(13q21) com
CT 848	SCC	16	17q12 and Episomal	HNF1B	-
CT 914	SCC	16	17q23.1	VMP1	FRA17B(17q23.1) com
CT 1097	SCC	16	18q11.2	-	-
CT 740	SCC	18	18q21.3	-	FRA18B(18q21.3) com
		16	Episomal	-	-
CT 906	SCC	16	19p13.3	-	FRA19B(19p13) com
CT 889	SCC	16	19q13.43 and Episomal	-	FRA19A(19q13) com
CT 918	SCC	16	20q11.21 and episomal	COX4I2	-

Sample ID	Histology	HPV type	Integration locus	Gene	Fragile site involved
CT 1170	SCC	16	20q11.21	COX4I2	-
		18	-	-	-
CT 839	SCC	16	20q13.13	-	-
CT 1015	SCC	16	-	-	-
CT 1082	SCC	16	-	-	-
CT 1101	SCC	16	-	-	-
CT 1107	SCC	16	-	-	-
CT 1108	SCC	16	-	-	-
CT 1135	SCC	16	-	-	-
CT 1161	SCC	16	-	-	-
CT 759	SCC	16	-	-	-
CT 787	SCC	16	-	-	-
CT 792	SCC	16	-	-	-
CT 800	SCC	16	-	-	-
CT 828	SCC	16	-	-	-
CT 868	SCC	16	-	-	-
CT 887	SCC	16	-	-	-
CT 905	SCC	18	-	-	-
CT 934	SCC	16	-	-	-
CT 935	SCC	16	-	-	-
CT 951	SCC	16	-	-	-

Key: – SCC: Squamous cell carcinoma, AC: Adenocarcinoma, Com: Common Fragile site

## Discussion

It is proven beyond doubt that infection by HPV plays a major role in the etiology of cervical cancer. Reports from different parts of the sub-continent indicate a prevalence of HPV ranging from 73–99% [18]–[30]. In the present study we report 95% HPV positivity using three different primer sets. It is apparent from this study that a single set of primers is not sufficient to estimate the true HPV infectivity. Of the various HPV genotypes HPV16 was most common (60%), followed by infection with HPV18 alone (2%). Dual infection with HPV16/18 (6%) and HPV16/45 (3%) was also observed. These results are in concordance with other studies from the Indian subcontinent which reports 57–65% HPV16 positivity, followed by HPV18, 45, and 33 in cervical neoplasia [19], [20], [29].

Integration of the virus is common in late stage cervical cancers and is considered an important event in the progression of the disease. Integration generally occurs downstream of the early genes E6 and E7, often in the E1 or E2 region. The E2 gene is transcriptionally inactivated once the virus gets integrated due to disruption of its open reading frame. Viral E2 gene has been extensively studied and is known to play a role in viral replication as well as negative regulation of E6 and E7 genes [31].

Various techniques have been used to study integration of the virus, such as Ligation-mediated PCR [32], Restriction site-PCR [15] and APOT assay [6]. In order to limit our study to integration sites with a transcriptionally active viral genome, APOT assay was chosen. Also, APOT assay allows detection of integrated viral genome in clinical lesions even in the presence of a large excess of nonintegrated episomal form of viral genomes [6], [33]. The frequency of viral integration into the host genome in cervical carcinomas has been reported to be as high as 100% in HPV18^+^ tumors [34] and up to 80% in HPV16^+^ tumors [35]. In our study we found four HPV18^+^ samples where the virus was integrated and one HPV18^+^ sample where the virus was episomal. The incidence of integration in HPV16^+^ samples was higher. The mechanism of HR-HPV integration is not fully understood. It is speculated that integration might represent a chance occurrence, the probability of which increases with the frequency of double-strand breaks (DSBs) in host and viral DNA. Chromosomal fragile sites could represent the hotspots for HR-HPV integration.

The physical state and/or site of integration was studied in 86 cervical tumour samples infected with either or both of two HR-HPVs, HPV16 and HPV18, and with an adequate clinical follow-up. Episomal form of HPV was observed in 18 cases. Presence of only episomal form in these patients with advanced disease stage (predominantly FIGO stage IIIB), could indicate that either HPV integration is not solely responsible for the progression of the disease; or it could be a limitation of the technique resulting in failure of amplification of the integrant derived transcript. Recent studies have confirmed presence of only episomal form of the virus in advanced cervical squamous cell carcinomas [33], [36]. In addition, since APOT works on the basis of annealing of the Frohman primer to the polyA tail, cases in which polyA tail is located at a great distance from the forward primer, might not be amenable to amplification by PCR.

In the present study, we observed 12 samples where both integrated as well as episomal forms of the virus were present. In such cases, E2 may be available in *trans* to modulate the expression levels of oncogenic E6 and E7 [37]. Also according to the report by Pett *et al*., loss of episomes is as much important as integration of the virus into the host genome for progression of lesions to cervical neoplasia [38]. It would be interesting to see the expression of E2 and E6/E7 in cases where HR-HPV is present in episomal as well as integrated form or in samples where HPV is only episomal.

There are reports that HR-HPV proteins other than E6/E7 induce chromosomal instability and transformation [39]. It is reported that E2 stabilizes Skp2, an oncogene and this could lead to activation of S-phase entry [39]. Hamid et. al., [40] have also suggested a role for E2 in cell proliferation. Since cells in S-phase are more responsive to radiation, cancers with episomal E2 could be more responsive to radiation treatment. Comparison of the physical state of the virus (episomal/integrated) with the clinical outcome after radical radiotherapy revealed that patients with episomal form of the virus had increased disease free survival compared to those with integrated form. This observation is supported by various reports which state that the integration event is associated with a decreased disease free survival [41], [42]. However, there are contrasting reports as well, according to which physical state of the virus does not correlate with disease free survival [43], [44]. This needs to be studied further.

Although integration sites were distributed throughout the genome in different samples, there was a preference for certain chromosomal loci such as 1p, 3q, 6q, 11q, 13q, 6q and 20q. Certain specific regions in some of these loci such as 1p36.23, 1p36.33, 3q26, 3q28 and 20q11.21 showed repeated integrations, indicating that integration might not be a random event. Reports from studies involving western populations indicate that integration of the virus occurs most commonly at 8q chromosomal locus [3], [45], [46]. Integration at the 8q locus in our study was observed in only 1 out of 48 cases. This may be due to the difference in the ethnicity of the two populations.

The 3q, 13q and 20q loci besides being preferential target for HPV integration have been reported to be sites for genomic instability associated with cervical cancer. Gain of 3q and 20q, while loss of 13q has been reported in various stages of the disease [47]–[49]. Also more recent reports show that a significant association exists between genomic rearrangement and HPV integration [46]. It would therefore be interesting to study whether the preferential integration of the virus into these loci has a role to play in inducing genomic instability.

Most of the integrations (28/48) were found to be located within or near certain genes. This could indicate that the virus prefers transcriptionally active regions for the integration event. Such genes included oncogenes such as myc, transcription factors like TP63, MECOM, etc. This observation is supported by previous report by Wentzensen *et al* wherein they have shown involvement of tumour related genes (myc and TP63) in HPV integration process [50]. Studies in our lab have shown that some of the genes within which integration was observed, such as ABCB10, SLC25A36, IL8, COX4I2, HNF1B, myc, demonstrated increased expression (unpublished data), thereby indicating that upon integration within or near a particular gene the virus may bring about changes in gene expression.

Integration of the virus near or within fragile sites has frequently been reported [15], [45], [50]–[52]. Fragile sites are specific regions in the chromosomes that nonrandomly undergoes break in response to certain stress. This makes genes in or near these sites susceptible to foreign DNA integration. In our study 29/48 integrations were located within or near a common or rare fragile site which is in concordance with previous reports. Another observation of our study was that patients with viral integration at chromosomal loci 3q, 13q and 20q showed the worst prognosis amongst all. Whether this can have any clinical implication in prognosis of cervical cancer would be interesting and challenging to study.

## Supporting Information

Figure S1 **Kaplan-Meier analysis for two HR-HPV types – HPV16 and/or HPV18 and disease outcome.** Kaplan-Meier survival analysis for HPV16 and/or HPV18 in 125 patients who had a good clinical follow up was carried out. Patients with HPV16 infection alone showed a trend towards better disease free survival as compared to HPV18 infection alone and dual infection with HPV16/18.
(TIF)

Figure S2 **Genomic DNA PCR of the recurrent integration sites.** Representative gel images (a, b) showing HPV integration at the genomic level. Recurrent integrations at chromosomal loci 1p36.23, 3q28, 6q23.3, 8p11.21 and 11q13.1 is depicted.
(TIF)

Table S1 **Primer sequences.**
(DOCX)

Table S2 **Clinicopathological data for all cases where the viral integration was studied.**
(DOCX)

Table S3 **Sequence of HPV integration sites in the genome in 68 cases.** a) Integration sites for 48 cases with a high prediction score. b) Integration sites for 20 cases with a low prediction score.
(DOCX)
