# Supplementary figures and images for: HPV Genotyping and Site of Viral Integration in Cervical Cancers in Indian Women
Source: PLoS One. 2012 Jul 16;7(7):e41012. doi: 10.1371/journal.pone.0041012 (PMC3397968; doi:10.1371/journal.pone.0041012)

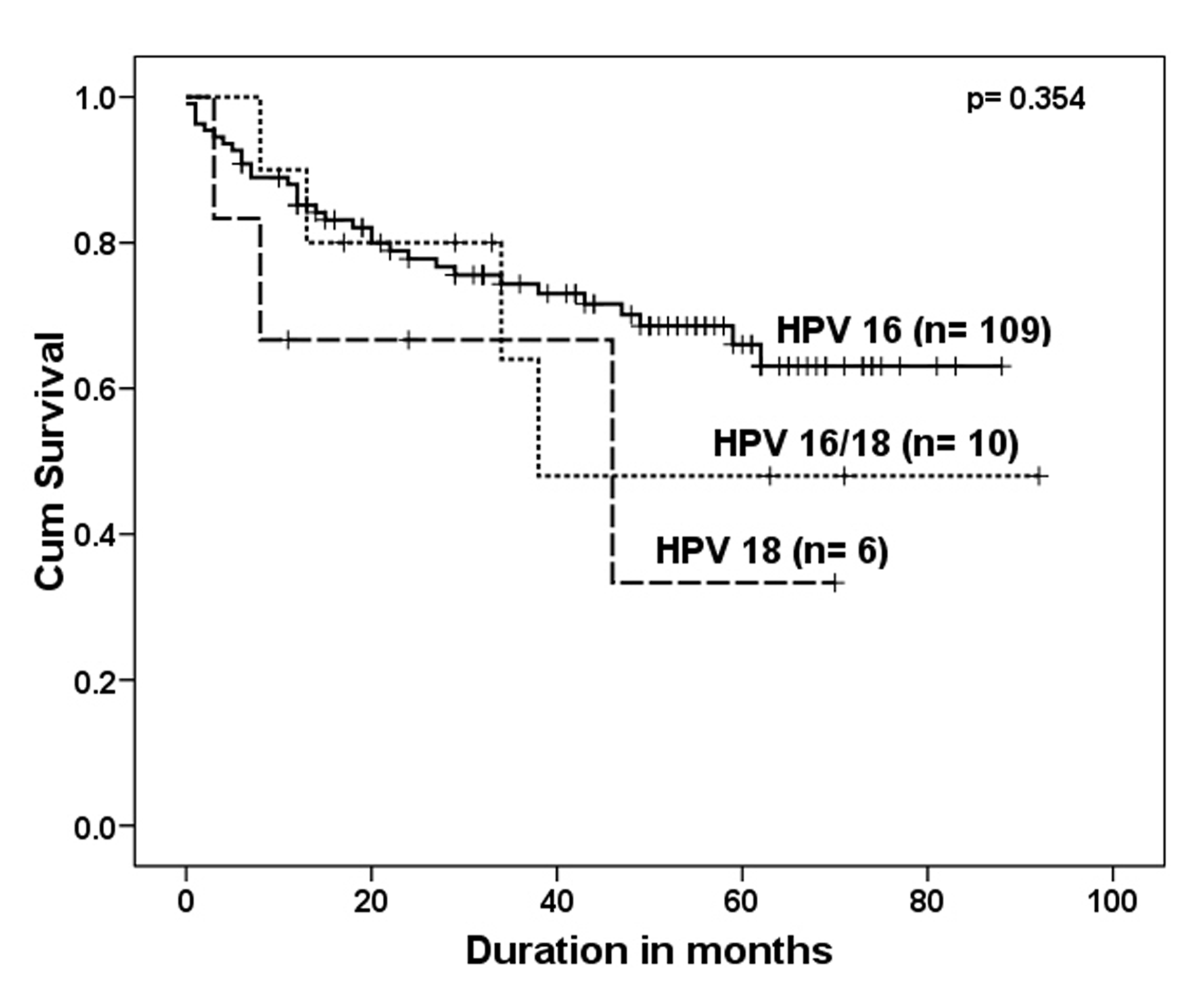

Supplement: Figure S1 — Kaplan-Meier analysis for two HR-HPV types – HPV16 and/or HPV18 and disease outcome. Kaplan-Meier survival analysis for HPV16 and/or HPV18 in 125 patients who had a good clinical follow up was carried out. Patients with HPV16 infection alone showed a trend towards better disease free survival as compared to HPV18 infection alone and dual infection with HPV16/18. (TIF) [file pone.0041012.s001.tif]

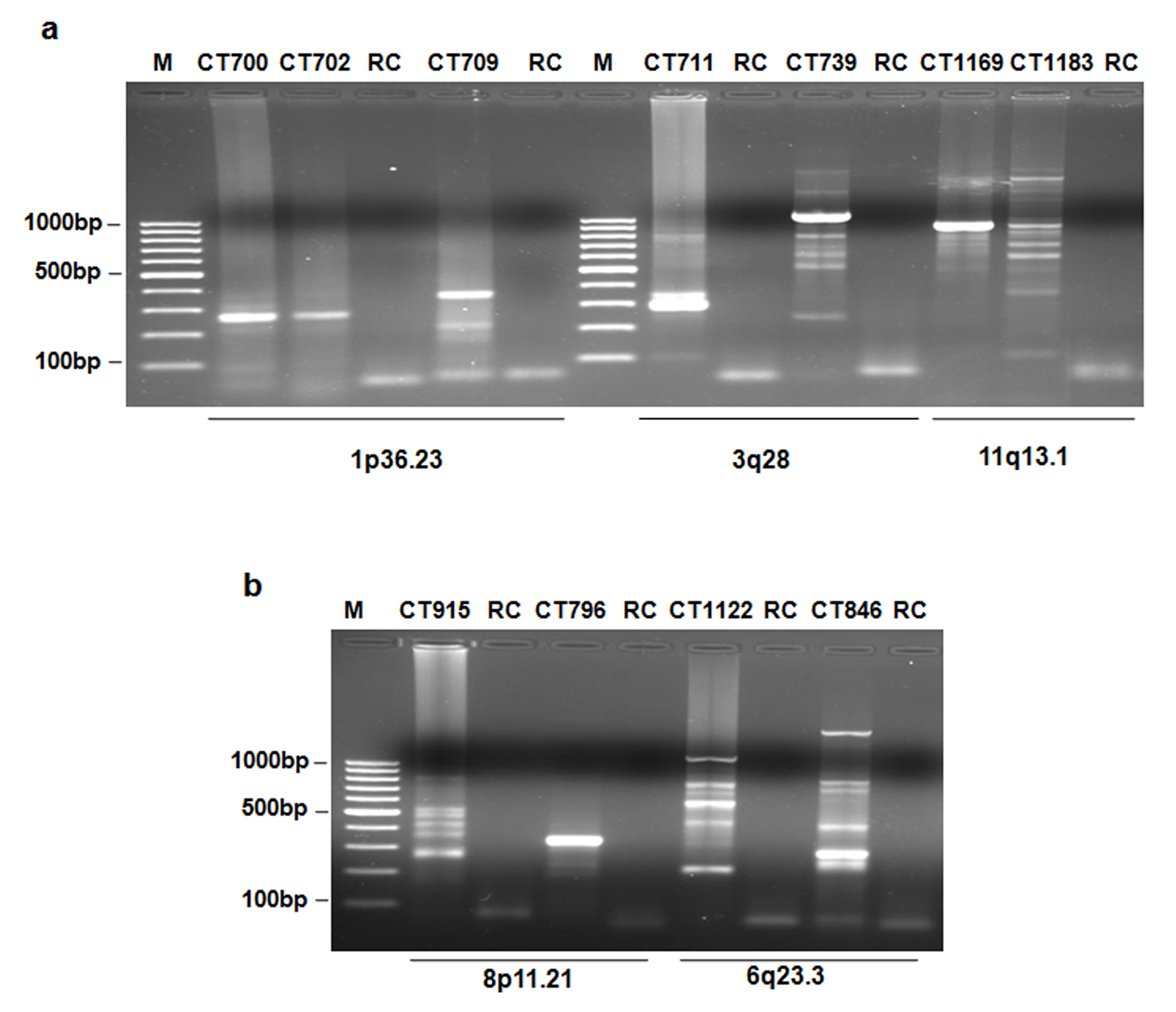

Supplement: Figure S2 — Genomic DNA PCR of the recurrent integration sites. Representative gel images (a, b) showing HPV integration at the genomic level. Recurrent integrations at chromosomal loci 1p36.23, 3q28, 6q23.3, 8p11.21 and 11q13.1 is depicted. (TIF) [file pone.0041012.s002.tif]
