# Supplementary material for: HPV Genotyping and Site of Viral Integration in Cervical Cancers in Indian Women
Source: PLoS One. 2012 Jul 16;7(7):e41012. doi: 10.1371/journal.pone.0041012 (PMC3397968; doi:10.1371/journal.pone.0041012)
Supplement: Table S1 — Primer sequences. (DOCX) [file pone.0041012.s003.docx]

Table S1. Primers used in the study

|  | Forward primers |  | Reverse primers |
| --- | --- | --- | --- |
| MY09 | 5’-CGTCCMARRGGAWACTGATC-3’ | MY11 | 5’-GCMCAGGGWCATAAYAATGC-3’ |
| HPV16 | 5’-AAGGCCAACTAAATGTCAC-3 |  | 5’-CTGCTTTTATACTAACCGG-3’ |
| HPV18 | 5’-ACCTTAATGAAAAACCACGA-3’ |  | 5’-CGTCGTTTAGAGTCGTTCCTG-3’ |
| p1-HPV16 | 5’-CGGACAGAGCCCATTACAAT-3’ | p3 | 5’-GACTCGAGTCGACATCG-3’ |
| p1-HPV18 | 5’-TAGAAAGCTCAGCAGACGACC-3’ |  |  |
| p2-HPV16 | 5’-CCTTTTGTTGCAAGTGTGACTCTACG-3’ | (dT).17-p3 | 5’-GACTCGAGTCGACATCGATTTTTT  TTTTTTTTTTT-3’ |
| p2-HPV18 | 5’-ACGACCTTCGAGCATTCCAGCAG-3’ |  |  |
