# Supplementary material for: HPV Genotyping and Site of Viral Integration in Cervical Cancers in Indian Women
Source: PLoS One. 2012 Jul 16;7(7):e41012. doi: 10.1371/journal.pone.0041012 (PMC3397968; doi:10.1371/journal.pone.0041012)
Supplement: Table S2 — Clinicopathological data for all cases where the viral integration was studied. (DOCX) [file pone.0041012.s004.docx]

Table S2. Clinicopathological data for all cases

| Sample | Stage | Age | Integrated/ Episomal | Disease free | Time taken to recur | Type (Recurrence/ NED) |
| --- | --- | --- | --- | --- | --- | --- |
| CT 702 | IIb | 56 | Integrated | Yes |  | NED |
| CT 777 | IIa | 78 | Integrated and Episomal | Yes |  | NED |
| CT 700 | IIIb | 61 | Integrated | Yes |  | NED |
| CT 1123 | IIIb | 45 | Integrated | Yes |  | NED |
| CT 709 | IIIb | 32 | Integrated | Yes |  | NED |
| CT 809 | IIIb | 70 | Integrated | Yes |  | NED |
| CT 712 | IIIb | 68 | Integrated | Yes |  | NED |
| CT 1138 | IIb | 40 | Integrated | Yes |  | NED |
| CT 1194 | IIIb | 55 | Integrated | Yes |  | NED |
| CT 866 | IIIb | 41 | Integrated | Yes |  | NED |
| CT 864 | IIIb | 44 | Integrated | Yes |  | NED |
| CT 733 | IIIb | 61 | Integrated | Yes |  | NED |
| CT 718 | IIIb | 57 | Integrated | Yes |  | NED |
| CT 739 | IIIb | 70 | Integrated | No | _ | LR |
| CT 892 | IIIb | 56 | Integrated | No | 16 months | PD |
| CT 785 | IIIb | 42 | Integrated | No | 30 months | LR |
| CT 755 | IIIb | 50 | Integrated | No | 3 months | LR |
| CT 1210 | IIIb | 52 | Integrated and Episomal | Yes |  | PD |
| CT 707 | IIb | 50 | Integrated | No | 7 months | LR |
| CT 723 | IIIb | 69 | Integrated | No | _ | DM |
| CT 706 | IIIb | 53 | Integrated | Yes |  | NED |
| CT 711 | IIIb | 56 | Integrated and Episomal | Yes |  | NED |
| CT 999 | IIIb | 53 | Integrated | Yes |  | NED |
| CT 1114 | IIIb | 42 | Integrated | Yes |  | NED |
| CT 819 | IIIb | 55 | Integrated | No | 1 month | PD |
| Sample | Stage | Age | Integrated/ Episomal | Disease free | Time taken to recur | Type (Recurrence/ NED) |
| CT 959 | IIb | 58 | Integrated and Episomal | Yes |  | NED |
|  |  |  |  |  |  |  |
| CT 1122 | IIIb | 42 | Integrated | Yes |  | NED |
| CT 846 | IIb | 39 | Integrated | Yes |  | NED |
| CT 1117 | IIIb | 54 | Integrated | Yes |  | NED |
| CT 893 | IIb | 62 | Integrated | Yes |  | NED |
| CT 1019 | IIb | 58 | Integrated | Yes |  | NED |
| CT 912 | IIIb | 56 | Integrated and Episomal | No | 35 months | DM |
| CT 796 | IIIb | 56 | Integrated | Yes |  | NED |
| CT 915 | IIIb | 57 | Integrated | No | 5 months | LR |
| CT 1160 | IIIb | 64 | Integrated | No | 2 months | LR+DM |
| CT 889 | IIIb | 45 | Integrated and Episomal | No | 12 months | LR+DM |
| CT 1162 | IIIb | 67 | Integrated and Episomal | Yes |  | NED |
| CT 714 | IIIb | 72 | Integrated | Yes |  | NED |
| CT 753 | IIIb | 40 | Integrated | Yes |  | NED |
| CT 1202 | IIIb | 50 | Integrated | Yes |  | NED |
| CT 821 | IIb | 87 | Integrated | Yes |  | NED |
| CT 859 | IIIb | 48 | Integrated | Yes |  | NED |
| CT 837 | IIIb | 40 | Integrated | No | 2 months | PR |
| CT 716 | IIIb | 56 | Integrated | Yes |  | NED |
| CT 1169 | IIIb | 54 | Integrated and Episomal | Yes |  | NED |
| CT 836 | IIIb | 58 | Integrated | Yes |  | NED |
| CT 871 | IIa | 46 | Integrated | Yes |  | NED |
| CT 1183 | IIIb | 70 | Integrated | Yes |  | NED |
| CT 744 | IIIb | 50 | Integrated | No | 9 months | DM |
| CT 896 | IIIb | 45 | Integrated | No | 44 months | DM |
| CT 976 | IIIb | 35 | Integrated | No | 39 months | DM |
| Sample | Stage | Age | Integrated/ Episomal | Disease free | Time taken to recur | Type (Recurrence/ NED) |
| CT 763 | IIIb | 60 | Integrated | No | 19 months | LR |
| CT 927 | IIIb | 44 | Integrated | No | 4 months | DM |
| CT 1094 | IIIb | 58 | Integrated | No | 3 months | DM |
| CT 793 | IIIb | 38 | Integrated | Yes |  | NED |
| CT 922 | IIIb | 53 | Integrated | No | 12 months | LR+DM |
| CT 940 | IIIb | 60 | Integrated | Yes |  | NED |
| CT 1215 | IIIb | 55 | Integrated | No | 1 month | LR |
| CT 914 | IIb | 61 | Integrated | Yes |  | NED |
| CT 848 | IIIb | 68 | Integrated and Episomal | Yes |  | NED |
| CT 1097 | IIIb | 60 | Integrated | Yes |  | NED |
| CT 825 | IIIb | 45 | Integrated | Yes |  | NED |
| CT 740 | IIIb | 39 | Integrated and Episomal | No | 11 months | DM |
| CT 906 | IIIb | 62 | Integrated | Yes |  | NED |
| CT 752 | IIIb | 59 | Integrated and Episomal | Yes |  | NED |
| CT 839 | IIIb | 70 | Integrated | No | 26 months | DM |
| CT 785 | IIIb | 42 | Integrated | No | 30 months | LR |
| CT 1170 | IIIb | 60 | Integrated | Yes |  | NED |
| CT 918 | IIIb | 45 | Integrated and Episomal | Yes |  | NED |
| CT 1161 | IIIb | 40 | Episomal | Yes |  | NED |
| CT 951 | IIIb | 54 | Episomal | Yes |  | NED |
| CT 1108 | IIa | 54 | Episomal | Yes |  | NED |
| CT 887 | IIIb | 51 | Episomal | Yes |  | NED |
| CT 934 | IIIb | 44 | Episomal | Yes |  | NED |
| CT 1135 | IIIb | 46 | Episomal | Yes |  | NED |
| CT 868 | IIIb | 60 | Episomal | Yes |  | NED |
| CT 1107 | IIIb | 55 | Episomal | Yes |  | NED |
| CT 828 | IIb | 66 | Episomal | Yes |  | NED |
| Sample | Stage | Age | Integrated/ Episomal | Disease free | Time taken to recur | Type (Recurrence/ NED) |
| CT 800 | IIb | 56 | Episomal | Yes |  | NED |
| CT 1015 | IIb | 49 | Episomal | Yes |  | NED |
| CT 1082 | IIIb | 55 | Episomal | Yes |  | NED |
| CT 905 | IIIb | 49 | Episomal | No | 46 months | LR |
| CT 787 | IIb | 45 | Episomal | Yes |  | NED |
| CT 935 | IIb | 67 | Episomal | Yes |  | NED |
| CT 792 | IIIb | 60 | Episomal | No | 2 months | LR |
| CT 759 | IIIb | 65 | Episomal | Yes |  | NED |
| CT 1101 | IIB | 56 | Episomal | Yes |  | NED |

Key:- NED: No Evidence of Disease; LR: Locoregional Metastasis; DM: Distant Metastasis; PR: Persistent disease; PD: Progressive disease
