# Supplementary material for: HPV Genotyping and Site of Viral Integration in Cervical Cancers in Indian Women
Source: PLoS One. 2012 Jul 16;7(7):e41012. doi: 10.1371/journal.pone.0041012 (PMC3397968; doi:10.1371/journal.pone.0041012)
Supplement: Table S3 — Sequence of HPV integration sites in the genome in 68 cases. a) Integration sites for 48 cases with a high prediction score. b) Integration sites for 20 cases with a low prediction score. (DOCX) [file pone.0041012.s005.docx]

|  |
| --- |

**Table S3a. Sequences of site of viral integration with high score**

| **Sample ID** | **Chromosomal loci** | **Sequence** |
| --- | --- | --- |
| CT 700 | 1p | CTTTTCGGTTGTGCGTACAAGCACACACGTAGACTTTCGTACTTTGGAAGACCTGTTAATGGGCACACTAGGAATTGTGTGCCCCATCTGTTCTCAGAAACCATAATCTACCATGGCTGATCCTGCAG  ATCCCCTATTGGACGAACCCTCCTTTACCCCTGACTGACTCACCAGTCCCTAATGCTGGTCAGATGTCCTGAACAAGGAAAGCCGTGATTTCTGCGACCAGAGAGAATCGGTCAGTCACGGTGTCCCTCAGCCAAAATGAACTCATCCTATTGTCTCCGGAGAGCTGGTAAATCTGCAGCTGAAAAACATACAGTTCTGTGTTTCAAAATGCAAAAACGCACATTTCCCTGACCTCGTGCTCCTGACATTCCCAACCAATGACTATAAATCTGCTCCTGGATTGTGAATTGAAATTAAACCAA |
| CT 702 | 1p | CTTCGGGTTGTGCGTACAAGCACACACGTAGACATTCGTACTTTGGAAGACCTGTTAATGGGCACACTAGGAATTGTGTGCCCCATCTGTTCTCAGAAACCATAATCTACCATGGCTGATCCTGCAG  ATCCCCAGTTGGAGGAACCATCCTTTACAGCTGACTGACTCACCAGTCCCTAATGCTGGTCAGATGTCCTGAACAAGGAAAGCCGTGATTTCTGCGACCAGAGAGAATCGGTCAGTCACGGTGTCCCTCAGCCAAAATGAACTCAGCCTATTGTCTCCGGAGAGCTGGTAAATCGGCAGCTGAAGAAACATACAGTTCTGTGTTTCAAAATGCAAAAGACCACATTTCCCTGACCTCGGGTGCTCGCTGACACATTCCCAACCAATGACTATAAATCTGCTCCTGGATTGTGGTAAGTTGAGAAATTAAACACAAATATCCCATCTGATAAACAA |
| CT 709 | 1p | TKTTKSGWAAGCAMACACGTAGACATTCGTACTTTGGAAGACCTGTTAATGGGCACACTAGGAATTGTGTGCCCCMTCTGTTCTCAGAAACCATAATCTACCATGGCTGATCCTGCAGGTMCCAATGGGGAAGARGGTTCKGTTTGTAATG  AYTGACTCACCAKTCCCTRMTGCTGGKCAAATGTCCTACACAAGGAAAGACGGACTTTATTCCTTATATAAAMTATGCRWCGKATTTCGTGTCCCTCARCCAAAATGAACTCWTCCTATTGTCTCCGGAGAATGGTAAATCGGYAGCTGAAKAAACWTACAKTTCTGTGTTTCCCCTGTAAAASAYGRCATTTCCCTGACCTCGGGTGCTCGCTGACGCATTCCCTYCCAATGAMTACCACTCTGCTCCTGGATTGTGGCCCSTTGASA |
| CT 712 | 1p | CTTCGGTTGTGCGTACAAGCACACAAACGGTATACATTCGTACTTTGGAAGACCTGTTAATGGGCACACTAGGAATTGTGTGCCCCATCTGTTCTCAGAAACCATAATCTACCATGGCTGATCCTGCAG TCCCCCCAGCACCGAGTTTCCTCCAGCAGATTCGGGGCTCCCTCCTGCCAGCTTCTGCTCAGATCATGATTGGCGCCTATCCCACAGATGGTGATCAGAAGACCTATTGTGTGCCCGACACTGGATGAGGCATTTTATAGTCAGGGACAACATGACCGCTTGACTCGCCTAAAAAAGAAAAAAAAAAGTTCTAAGCCTAGGATCTGGGGCGTGAGGTGTGTGTTATGGGAGGAGGCTGGTAGGGGAGGTGGTTCCTTTATTATGTTATTTGGAGGAAAAAAGTCGGGGAATACTATATATTTTGCGTGAACCCCCCCTTTTAAATTGGGTGGGGCTCAAAAA |
| CT 809 | 1p | AKKSSGTWMAAGCACACACGTAGACATTCGTACTTTGGAAGACCTGTTAATGGGCACACTAGGAATTGTGTGCCCCATCTGTTCTCAGAAACCATAATCTACCATGGCTGATCCTGCAGGTGTATTARCTGTCAAAAGCCACTGTGTCCTGAARAAAAGCAAAGACATCTGGACAAAARGMAAAGATTCCATAATATAAGGGGTCGGTGGACCGGTCGATGTATGTCTTGTTGCAGATCATCAAGAACACGTAGAGAAACCCAGCTGTAATCATGCATGGAGATACACCTACATTGCATGAATATATGTTAGATTTGCAACCAGAGACAACTGATCTCTACTGTTATGAGCAATTAAATGACAGCTCAGAGGAGGAGGATGAAATAGATGGTCCAGCTGGACAAGCAGAAYCGGACAGAGCCCATTACAAT  CTCCAGCATTCCCCCTCAAACCTAAAAAAAAA |
| CT 866 | 1p | CCTTCGGTTGTGCGTACAAGCACACACGTAGACCTTCGTACTTTGGAAGACCTGTTAATGGGCACACTAGGAATTGTGTGCCCCATCTGTTCTCAGAAACCATAATCTACCATGGCTGATCCTGCAGGTGTATTAACTGTCAAAAGCCACTGTGTCCTGAAGAAAAGCAAAGACATCTGGACAAAAAGCAAAGATTCCATAATATAAGGGGTCGGTGGACCGGTCGATGTATGTCTTGTTGCAGATCATCAAGAACACGTAGAGAAACCCAGCTGTAATCATGCATGGAGATACACCTACATTGCATGAATATATGTTAGATTTGCAACCAGAGACAACTGATCTCTACTGTTATGAGCAATTAAATGACAGCTCAGAGGAGGAGGATGAAATAGATGGTCCAGCTGGACAAGCAGAATCGGACAGAGCCCATTACAAT  CTCCAGCATTCCCCCTCAAACCTAAAAAAAA |
| CT 1138 | 1p | CTTTCGGATTGTGCGTACAAGCACACACGTAGCCATTCGTACTTTGGAAGACCTGTTAATGGGCACACTAGGAATTGTGTGCCCCATCTGTTCTCAGAAACCATAATCTACCATGGCTGATCCTGCAGGTGTATTAACTGTCAAAAGCCACTGTGTCCTGAAGAAAAGCAAAGACATCTGGACAAAAAGCAAAGATTCCATAATATAAGGGGTCGGTGGACCGGTCGATGTATGTCTTGTTGCAGATCATCAAGAACACGTAGAGAAACCCAGCTGTAATCATGCATGGAGATACACCTACATTGCATGAATATATGTTAGATTTGCAACCAGAGACAACTGATCTCTACTGTTATGAGCAATTAAATGACAGCTCAGAGGAGGAGGATGAAATAGATGGTCCAGCTGGACAAGCAGAATCGGACAGAGCCCATTACAAT  CTCCAGCATTCCCCCTCAAACCTAAAAAAAAA |
| CT 864 | 1q | CTTCGTTGTGCGTACAAGCACACACGTATCCATTCGTACTTTGGAAGACCTGTTAATGGGCACACTAGGAATTGTGTGCCCCATCTGTTCTCAGAAACCGAGGACTAACATGGCTGATCCTGCA  AGTGTATCAACTGTCAACCACCTGAGTGTTGTGATAATTCGCTTTACACCCTGGTGATATCAACAGCCCCCTAAGCTTTCGGGGCAACTGGTAGATGAAAATGTATGCCCCGATCTGCTCTCCTCCAACGTGCAAACCTACTCTAAATCCCAAATCACTACCCAGGAGGGTCCTTTCTATGACAATGGATTTGATTTAAAAAATGATTTTCTGGAGTTGCACCGGAAAACCTCACTAGCCCCACACGCCCACTTCCACAGCCCACAGGTGCAGGTTTACCTCTATACATACGAATATTTCATACATGCTTCTCCCCACTAACCTAGAAGCCCCAGCAGAAGTGAGGACCATGTTTCCTTGTGCATGCCACTGTAAACCCAGGGCCCAGCCCAGAATGAGGGCTCAACAGATGTTTAAGAAGGAGTGAAGATTTAGAGAAATCGAAAAAATTTTAGATCTTACAAGGCCCTTGCATCTAAACACTCCTCCAATTATGGCTAAAAAATGAGGAGCTATAGAAAACTGTCCTATTTAAATTTTGTTTCTAAAAACATTCATTTTGGCTTCTTTTAAAAAAGTTACTCAATTACTGAACAGATCACTGGGTGTGGCCTGTTGAACACAGTGCTGTAACTTCCTGCACAGAGACAAGTCTGAAAGTTTTGCTTTCCGCTGTGAAAGCATTGGTGACTGCTTGACAGACTCGCTTGAAGCAATAATGTGTTTTTGGTTAAAAAAAAAAAAACAGAGTCATCACAAAAAAAAAAAAAATTTTTTTTTACACAATTGGAGGCGAACACG |
| CT 755 | 3q | CTTCGGTTGTGCGTACAAGCACACACGTCGACATTCGTACTTTGGAAGACCTGTTAATGGGCACACTAGGAATTGTGTGCCCCATCTGTTCTCAGAAACCATAATCTACCATGGCTGATCCTGCAG  ATGGAGTCTCCATGGTGCAATCATGGCTCACTGCAGCCTCAAACTCCTGGGCTCAAGCAATCCTCCCACCTCAGCCTCCCAGGTAGCTAGGATTATAGGCATGCATTTTTTATAGAGATTAAGTCTTGCAATGTTGCCCAGGCTGTTCTTAAACTCCTGGCCTCAAGCAATCCTCCTGCCCTGGCCTCTCAAAGCTCTGGGATTACAGGCATGATCCACTGCGCTCAGCCCTCAGCCTTATAAACCAGAATCCTCAAGTCAACCTTTCATTATTACCCCACCCCTATCCAATTCCCCAACCCCACAAGCCTAAGAATTGTGCCAGCTTGTGTTACAATTATAAGTGGAAAAAAATA |
| CT 785 | 3q | CTTCGTTGTGCGTACAAAGCAACACGTCCACATTCGTACTTTGGAAGACCTGTTAATGGGCACACTAGGAATTGTGTGCCCCATCTGTTCTCAGAAACCATAATCTACCATGGCTGATCCTGCAG  ATGGAGTCTCCATGGTGCACTCATGGCTCACTGCACCCTCAAACTCCTGGGCTCAAGCAATCCTCCCACCTCAGCCTCCCAGGTAGCTAGGATTATAGGCATGCATTTTTTATAGAGATTAAGTCTTGCAATGTTGCCCAGGCTGTTCTTAAACTCCTGGCCTCAAGCAATCCTCCTGCCCTGGCCTCTCAAAGCTCTGGGATTACAGGCATGATCCACTGCGCTCAGCCCTCAGCCTTATAAACCAAATCCTCAAGTCAACCTTTCATTATTACCCCACCCCTATCCAATTCCCCAACCCCACAAGCCTAAAATTGTGCCAGCTTGTGTTACAATTATAATGGAAAAAAAATAATATT |
| CT 723 | 3q | CGGKKKKKCGWMAASCACMCACGTAGACATTCGTACTTTGGAAGACCTGTTAATGGGCACACTAGGAATTGTGTGCCCCATCTGTTCTCAGAAACCATAA  TCTACCATGGCTGATCCTGCAGCAATTAAACTCCAAATGGTACTGCTGSMAGAASAACCCCTGRTGRCCCCRCTCTTCCTCCAAKACTCAATACATCCWCCKGAGAAGGASGMMCTGCTGCTCCTCTGCTGGCTCCTTTTTTTCAGGAAGAGGTGRCMAMRAATAATCRTCSWCCACCCCCCCMYARCAGTTRTTTTTTCTGCTCCTCAKGGGGGAAGAAATACWAGTGGTGCTTCTTAAAATTTTTTTATTARGAAAAAAAAGATAMAARGAGTACTTGTTGAKGMKTTTTTTTCTGTTAAAAAAAACCAACCAASAATTTCTTTTCTTAYAAAAAAAAAACTTGTTGGGSCGGCGGGSCAAGCTTTTATAATACRATAATGCCSCCCGTAAAAAATGGTCCCCCCAAWATGGCKACTCCCGTCTTCTTCTTGCCACGTGGGGTGACTGTCGTGGCGGCCACCTCAAAATAACACGTTGTSWTAACATCATGGCGACCAGTATTTTTTTCTTAATAAACAACTGAGGGAGG |
| CT 706 | 3q | TGGGKTKSSGWMAARGCACACACGTAGACATCCGTACGTTGGAAGACCTGTTAATGGGCACACTAGGAATTGTGTGCCCCATCTGTTCTCAGAAACCATAATCTACCATGGCTGATCCTGCAGCAGCGACRAAGTATCCTCTGCTGAAATTACTAGGCAGCACTTGGCCAACCACTCCGCCGCGACCCATACCAAAGCCGTCGCCTTGGGCACCAAAGAAACACAGACGACTATCCAGCGACCAAGATCAGAGCCAGACACCGGAAACCCCTGCCACACCAACAAGTTGTTGCACAGAGACTCCGTGGACAGTGCTCCAATCCTCACTGCAGTTAACAGCTCACACAAAGGACGGA  AGTGTGAAATGTGTCACTCACATAAAAGCCCCTGTTTAAAGAAAGGGKGGGACGGGGGAGTGCAGAGGAGTAACTATTTAGGGGTGATTTAAATATTTAGGTCTCCTTTTTTTCTCCTTCAAATTAATAMAAAGGCCTTCGKATATAGTTAARAGAKTTTGAAA |
| CT 892 | 3q | CTTCGGTGTGCGTACAGCACCACGTAGACATTCGTCCTTTGGAAGACCTGTTAATGGGCACACTAGGAATTGTGTGCCCCATCTGTTCTCAGAAACCATAATCTACCATGGCTGATCCTGCAGG  ATGAATAAAGCACGGAGAACCCGAGGGAGTGTGAAGATCACATCAGGTGTCTTTCGGTCAGTGGAGGAAACACCCGGAAGCAGTGAAACGCACTGGGCTTATTGCAACAACCCCAGCTACTGAAAATGCCACCCTCTCCACGACGAAGCACCGCCAGTCCCAAACTCCAATCAGCCGCTGGGCTGGGAGGCCGAAAAGGCAAGAAGACACAGAGCGGGGTTGAAATGAAGAAGGAACTCTATCTCAACGAGACTCATTACAGTATGCAAAACCTAAAAAACTGTACCCTGTGTTGTAATGAGAATATGACTGCATGGGAAATGTGGGTTACAACTCTTGACTTTACCTGCTTTTCACATATTTTCACATAGTGAGTAAAATCGCTGTGGGGAGGAAATGGGCAATGTGGAAATTTCTTCTTATTCCTCCCCTCCCAACTTTCACAACCCTCAGTGTCTATCTCCCCTCTTTGGTGTTCTCCCTCGGTCCGATGGGGTCTTGTGTCCCTGCGGTGCTCTCAAGATCGTTTATTTCGATGAAAAGACAAAAAAAAAAATCACTCTACATCCTACCTGTGAG |
| CT 1210 | 3q | CTTTCGGTTGTGCGTACAAGCACACACGTAGATCCTTCGTACTTTGGAAGACCTGTTAATGGGCACACTAGGAATTGTGTGCCCCATCTGTTCTCAGAAACCATAATCTACCATGGCTGATCCTGCAGGTATCCA  TGCTTGTTAGCCAAGGGGAAGTACAATGGGGCAATCTCCGCTCACCACAACCTCCGCCTCCCAGGTTCAAGCGATTCCCCTGCCTCAGCCTCCCAACTAGTTAGTATTACGGGCATGCGCCACCACGCCTGGCTAATTTTGTATTTTCATTAGAGACGGGGTTTCTCTGTGCTGGTCAGGCTGGTCTCAAACTCCCGACCTCAGGTGATCTGCCTGCTTCAGCCTCCCAAATTGCTGGGATAACAGGTGTGAGGCACCGTGCCTGGCCCTAAATGGCTTTTCTACACGTCAGTTTCTTCAATACATTTGGATTACAAAATTAATGGAAATATTTCTCTTGACGCTTGAACAGGCCAAAAACCCCCCTTTTT |
| CT 711 | 3q | GTTATATAAAAACCAAATACCTTCGGTTGTGCGTAACAAGCCACCGTAGACATTCGTACTATATAGGAAGACCTGTTAATGGGTCCCACTAGGAATTGTGTGCCCCATCTGTTCTCAGAAACCATAATCTACCATGGCTGATCCTGCAG  TCTGGTTATGAAGGTCGAGTCCCACTGAAGAGCGCTCGTCTGTTTTATGACATCCGCCAAAACGCTCAAAGGATTGTACAATCTTATTTTATGCTAAACTCAAATCAGTATTTTTCCTATACACACATGGTCTGCCAAACAACCCTGTCTGGTCACCAGGATATAATAAATGACCTCCTCCATCCCATCCATGCTGACAACTGTTTGTTGTATCCACACGCCAACAAATGCTATTAGGAGCCTCCTGCTTACTCCTTTCATAACTATATTGCTCTCCTATATATGAATGATGACTTTGAAGGAGGAGAATTCATATTCACAGAGATGGATGCTAAGACTGTGACTGCCTCTATAACATTGAACTGGACATGCTGGATCAATTTCTCATCTGGAGGAGAGAACCCTCATGGGGTGAAGGGAGTCACCAAAGGAAATTATGTCTGCTGTGGCTCTGTGGTTCACCTTGACCCCTTTATAAAAATTAAAAAA |
| CT 999 | 4q | CTTCGGTTGTGCGTACAAGCACACACGTAGACATTCGTACTTTGGAAGACCTGTTAATGGGCACACTAGGAATTGTGTGCCCCATCTGTTCTCAGAAACCATAATCTACCATGGCTGATCCTGCAGG  CCAAGATGACAACGAGCCCAGCTGAAGCTGACATCCCAGCAAATTGCATGACAAATTGCAAAGACGACTAACCCACAACCTACTCTTCTGGAAAATACAATTTAAATAAAATAATTTTAAGTGAAAAAAAAAAAAAAACCCCCTGTCCCAAAAAAAAAAAAAATTTTTTTTCTA |
| CT 1114 | 4q | CTTCGGTTGTGCGTACAAGCACACACGTAGATCTTCGTACTTTGGAAG  TTTTTTTTTTTTTTTTTATACAAAAGGATTTATTAAAAAACCAGTAAGACACTACTACATCATGACACTGTCACACTGGGCTTTTAACACAAGACTTGCTCTACAATACTGGGGGAAAGGGCATAAAACACAAATTGATTCTGAAGCATAGCAATTAAGAAATAAAACAATGAAAGCAAATTTCTTTTAATGAGAACTCAGAATTAAACTTCAGAGGGACCCAACGTCATACTTCCATTCAGGGACTTGATACAAAAAATTTAGTTTGAACTGCTATTAGCAGGTGGCAGGAGCCACCTTCAAATGAATCTTCAAATTGGAAAATACTGCTTCACCACCTGTTGGGGATAAGTTGCAAATGGAATAATTTAGTATGGTTTGTAGCTATTTTGATGACCACCTCGCCTGGATACCTTCCCATAACCAGTGCGGTACCACCCAATCGGATCCCGGGCCCGTCGACTGCAGAGGCCTGCATGCAAGCTTTCCCTATAGTGAGTCGTATTAGAGCTTGGCGTAATCATGGTCATAGCTGTTTCCTGTGTGAAATTGTTATCCGCTCACAATTCCACACAACATACGAGCCGGAAGCATAAAGTGTAAAGCCTGGGGTGCCTAATGAGTGAGCTAACTCACATTAATTGCGTTGCGCTCACTGCCCGCTTTCCAGTCGGGAAACCTGTCGTGCCAGCTGCATTAATGAATCGGCCAACGCGCGGGGAGAGGCGGTTTGCGTATTGGGCGCTCTTCCGCTTCCTCGCTCACTGACTCGCTGCGCTCGGTCGTTCGGCTGCGGCGAGCGGTATCAGCTCACTCAAAGGCGGTAATACGGTTATCCACAGAATCAGGGGATAACGCAGAAAGAACATGTGAGCAAAAGGCCAGCAAAAGGCCAGGAAACCGTAAAAAGGCGC |
| CT 1122 | 6q | CCTTCGGGTTGTGCGTACAAGCACACACGTCGACATCCGTACAGTTGGAAGACCTGTTAATGGGCACACTAGGAATTGTGTGCCCCATCTGTTCTCAGAAACCATAATCTACCATGGCTGATCCTGCAGG  CTCAACTGGTGGAAACGGTATGCAAGCGGGGGCAAATCATGAGGAAAAAACTCTCTCACCATGCATAGAATCATCTTCACCCCATTCTCAAGCACGTGCATAACTAAATATGTACATGTGAGGCTACACTGATCCACCCCATGCACCAGTTACACCATCATCTTGATGCAATGCATCCCCCTGGACCGTGCCCTAATGGTTTCCCCTTCAATTCGCTACTTGAAAGGGCGGATTACCTGTAAGACTCCCACTACACCCATAGTATTCTTTAAGGTGATGCTAATTCTTTAAAATGTTTAGATACAAATGCATAAAGATTGTAAATTGTGTCCATCTTTGTCGTCCAATGGCATTGGACAGGACTTAATGTAAATATACTTTGCATTGTTACACTTCTATGGGCATAACATTTTTAGAAAACCAA |
| CT 846 | 6q | CTTCGTTGTGCGTACAAGCACACACGTAGCCATCCGTACGTTGGAAGACCTGTTAATGGGCACACTAGGAATTGTGTGCCCCATCTGTTCTCAGAAACCATAATCTACCATGGCTGATCCTGCAG  AGAATGTAAGCTCCATAAGAGCAGGAACCTTAACAGCCTTGCTGACCAATGAATCTCCGGTACTTGAATCATCAGAATACCTGACACGCTGCAGGTGATTATGAAATACTTGTGAAATGAAGAAAGAATGCATCCACTTCCTACCAAGAACCAGGCTGAGATAACTGGTAAATACGTCAGTTCATACAAGATACAAATAATCTTTTAACTTCCTTCAGTGATCTAAACTCTGATCTTGATAAAACTATATTACTAGAAACTACATCCTTGGACCACCTTAAACAAGTGAATTGCATATCACTTTCACTGTGTTACATTATTCACTGTGTTACATCTTAATGTTATTGGAGTTCACAATTCATAAAAAAGGGGGG |
| CT 1117 | 6q | CTGAACGTACAAAGCACACCGTATCTCATTCGTACTTATAGGAAGACCTGTTAATGGGCACACTAGGAATTGTGTGCCCCATCTGTTCTCAGAAACCATAATCTACCATGGCTGATCCTGCAG  ATTGATACTGACACATAAAGAAGTTAACCTGATGCAGCATCTGAGTAAAACACAGTGTAATTATAGAACAGCAGACCCAACCATCAAATACATAATATGTTATAAACATAGTTTAATTCTCATCAACTAGAAAGTA |
| CT 893 | 6q | CTTCGGTTTGTAGCGGTACAAAGCACACACGTATCCATTCGTACTTTGGAAGACCTGTTAATGGGCACACTAGGAATTGTGTGCCCCATCTGTTCTCAGAAACCATAATCTACCATGGCTGATCCTGCAG  ATTGATACTGACACATAAAGAAGTTAACCTGATGCAGCATCTGAGTAAAACACAGTGTAATTATAGAACAGCAGACCCAACCATCAAATACATAATATGTTATAAACATAGTTTAATTCTCATCAACTAAAAGTA |
| CT 1019 | 7q | GKKKKCSGWMAAAGCAMACACGTAGACATTCGTACTTTGGAAGACCTGTTAATGGGCACACTAGGAATTGTGTGCCCCMTCTGTTCTCAGAAACCATAATCTACCATGGCTGATCCTGCAGGT  TTCCCACCTCCACTGCTTAAGGTTTAACTTAACAACTGACCTTCGACTGTAAAATGAAGCAAAGTATCTGCACTCAAGTAGTGAAAACCTAAGAAGTACTATTCTGCGCAACTTCCACAAGAGGGCAGTAGAACCCCTGAAGTAGTCCACAAAAGTAACAGACCTTGATTTTTCCTCTAATTTATTTTCATGCTAGCCTTCCTTAAAATCTTTTCCTAGAAAGCACTAACTAGCTGTGACCTGGCTAGGCCTCGTGGCTCATGCCTATCATCTCAGCACTTTGAGGGCCAAGGTGGGTGAATATCTTGAAGCCAGGAATTCAAGACCAGCCTGGACAAGATTGCAAGATCTTGTCTCTACAAAAAAATWAAATAAATTTTTTGGCTGGGAAAAAAAAAAAAAAAAAAAAAAAATTAAAATCCMCMKSWTCTCCCCTCCAAACTAAAAAAAAAAAAAATTAAKTAGATCATACAYAAAAAAATAAACAAAAATKASAAACTCTCACACTCGTGATGCGTGCGKWTTTACGTTGTTTGTCGWGAGTACTGAGCCCCG |
| CT 796 | 8p | CTTCGGTTGTGCGTACAGCACACACGTAGACATTCGTACTTTGGAAGACCTGTTAATGGGCACACTAGGAATTGTGTGCCCCATCTGTTCTCAGAAACCATAATCTACCATGGCTGATCCTGCAG  ATGAGTAAGAAAGGTCACTTCCTCGGCAGACCTCAGGTATCATTGGGGACTCAAAAAACAC |
| CT 915 | 8p | TATTCTTTCAAAAAGATGGAAGACCTGTTATCCGGGCACACTAGGAATTGTGTGCCCCATCTGTTCTCAGAAACCATAATCTACCATGGCTGATCCTGCAG  ATGAGTAAGAAAGGTCACTTCCTGGCAGACCTCAGGTATATTGGGGACATCAAGAAAAGAATAATTCATCAAAGTCTACAGATATTGCAGGCAAAGTTGAAACAAGCCAGACAAGTTCTCTTTGTATTCTTGAGAGACTTGGAAAGTCTAACCTGAGATTCCTTATTAAAAGTTCCAGCAAAACAGACTTA |
| CT 1160 | 8q | CTTCGTTGTGCGTACAAGCACACACGTAGCCATTCGTACTTTGGAAGACCTGTTAATGGGCACACTAGGAATTGTGTGCCCCATCTGTTCTCAGAAACCATAATCTACCATGGCTGATCCTGCAGG  CTCAAGATGATTAAGATGCTGAAATGACGAAGAAGAGTCATGAACACTGTTCTTCTCCAAGTGACAGACCTTCAAAATTTGTGGGGTTTTTTTTTTCAATTTCCTTTTGTTTGACTATGCCCACAAAATCCCTCAGGGAAAGGTCAGCAAAGGTCCAAATGTCAATCAATCATGAACAAAGGGGTAAAGTGCCAAGGACCTGACCCGTCAAACAATGACTCAAGGGGACAGTTCTTTACAAAACAGCAGGACCTGCCCTTGAAAATTCCAAATACAAAAGGTTCTACAGGGAAAAAAAAAAAAAAAAAAAAAAGACCCCCCCCCCACAAAA |
| CT 1162 | 9q | CTTCGGGTTGTGCGTAAAAGCACACACGTAGACATTCGTACTTTGGAAGACCTGTTAATGGGCACACTAGGAATTGTGTGCCCCATCTGTTCTCAGAAACCATAATCTACCATGGCTGATCCTGCAGCAACCAA  AGCATGGATAACCTAACGGTTTCCGGCATGAGCTGAACAAAGGAAACCGCCTACTGCCATACTGCTCTTCTAGGATTTTGCAAAGAAGAAAC |
| CT 714 | 9q | CTTCGGTTGTGCGACAAGAACCGTATACTCCGTACGTTGGAAGACCTGTTAATGGGCACACTAGGAATTGTGTGCCCCATCTGTTCTCAGAAACCATAATCTACCATGGCTGATCCTGCAGGT  CCTGAAACACTATAACACTGATGACTGATTTGGTGACTATTCTATCCCTGGCGTCTTCACAATGTCTGAATTTAGTATGTACTAATATACATTTGATGAATAATCAAACGCAAACAACC |
| CT 753 | 9q | TGGTGCTACAAACACCTATAATCCGTACGTTGGAAGACCTGTTAATGGCCACCTAGGATTGTGTGCCCCATCTGTTCTCAGAAACCATAATCTACCATGGCTGATCCTGCAG  AATCTTGCTCTGTCACCCAGGCTGGAGTTCTGAGAAGAATTGGCATTCAACCTCCCACCTATTGCACTGACCTCATTCATCACCCAAACCAGGACCCACATCCATCTGCATTCTCACTGCACAACCCGGCAATCCCTCTGCCGACACTATAATTCATTATCCCTACCACACCCCTCCAATCACGGTGATTGCCTGCATGAAGCCCGGCAATTCTGCAGGGAGGTGCAGGACTCACAAGCTCCAAATGAGGCTCAGCTATCACAGAATTCCATATCTGAGCTGGCTCTGGCTCTCAGTATGCTAAAAA |
| CT 821 | 10q | GAGGTCTGTTCCAAAAGGTAGCCGAGCGGGGAGGGCACGGGTTGTAACGGCTGGTTTTATGTACATGCTGGCAGCTCTAGATTATTAACTGTTGGTAATCC  ATATTTTAGGGTTCTGAGCTCTTTTGTTATTTTTATTATTTATCACTCTTTGGCTTGTCTTTTCACTCTTAATGGTATATATTGTACATCTTTTCTAGTTTGTTTTCAGTGAGAGGTTTCAGCACCATCTAGTCAACCA  CTGCCAGAGCAGAACTTGGTCTCCTATTTTAATGACTAAATTTTATTCCAGGCTGGGCATGGTGGCTCACGCCTGTAATCACAGCACTTTGGGAGGCTGAGGCAGGCAGATCACCTGAGGTCAGGAGTTCGAGACCAGCCTGACCACAATGGTGAAACCCCATTTCTACTTTTAAAAATACAAAATTTAGCCGGGCGTGATAGTGGGCTCCTGTGATCCCAGCTACTTAGGAGGCTGAGGCAGGAGAATCACTTGAACCGGGAAGCAAAAGATTGCAGTGAGCTTAGATTGCACCACTGCATTCCAGCCTGGGCAACAGAGGGAGACTTCATCTCAAAAAAAAAAAAAAA |
| CT 859 | 10q | GACGTTCATCTGAGTGTTTATTGGAAAAGCGTATCCCATTCGTACTTTGGAAGACCTGTTAATGGTCACACTAGGAATTGTGTGCCCCTTCTGTTCTCAGAAACCATA  TTCCCTTTGGAATGATCCTCCCCCCCACACCTAGTACTTTTCCCTCCCTTTTGTGAAGATCTTGACCAATGGCTAAGTGAAGATGACAATCATGTTGCACAATTCACTGTAAAGCTGGAAAGGGACAAACTGGTGTAATGATATGTGCATATTTATTACATCGGGGCAAATTTTTAAAGGCACAAGAGGCCCTAAATTTCTATGGGGAAGTAAGGACCAAAGACAAAAAGGTAAGTTATTTTTTGATGTTTTTCCTTTCCACTTCCTGGATCTGAAATTATTGAAAAA |
| CT 1169 | 11q | CTTCTCGAAGGAAAAAAAGACCTGTAATGGCCACCTAGAATTAAGTGTGCCCCATCTGTTCTCAGAAACCATAATCTACCATGGCTGATCCTGCAGG  ATGAATCTGAGGACACAGATACTGATGGGGAAGAGGAGACATCACAACCCCCACCCCAGGCCAGCCACCCCTCTGCCCACTTTCAGAGCCCCCCGACACCCTTCCTGCCCTTCGCCTCTACTCTGCCTTTGCCCCCAGCGCCCCCGGGCCCCTCAGCACCTGATGAAGAGGACGAAGAAGATTACGACTCCTAGCGCCTTCTGCCCCCCAGACCATAGCCCCTTTTAGTTGGTTTTAGTTGCTCTGGGGGGAGGAGAGAAGGTAGAGCTGTTCTTAAATTTATTAAAAAAAAAAAAAAAAAAAATCGATGTCGACTC |
| CT 1183 | 11q | CTCTTCGGTTGTGCGTACAAGCACACACGTACGACATTCGTACTTTGGAAGACCTGTTAATGGGCACACTAGGAATTGTGTGCCCCATCTGTTCTCAGAAACCATAATCTACCATGGCTGATCCTGCAGG  ATGAATCTGAGGACACAGATACTGATGGGGAAGAGGAGACATCACAACCCCCACCCCAGGCCAGCCACCCCTCTGCCCACTTTCAGAGCCCCCCGACACCCTTCCTGCCCTTCGCCTCTACTCTGCCTTTGCCCCCAGCGCCCCCGGGCCCCTCAGCACCTGATGAAGAGGACGAAGAAGATTACGACTC  CTAGCGCCTTCTGCCCCCCAGACCATAGCCCCTTTTAGTTGGTTTTAGTTGCTCTGGGGGGAGGAGAGAAGGTAGAGCTGTTCTTAAATTTATTAAAAAAAAAAAAAAAAAGGGATGGCGGCCCCGGCCAACAGATGTTCCCAGGGACCCAGCCTCAATTCACTGGGGGCGTTTTACACGGCGGGAAGGGGGAAAACTCGGAGGAATCCAACTTAGAGGCTATGGGAAAAA |
| CT 836 | 11q | CCTTCGGTTGTGCGTACAAGCACACACGTAGACATTCGTACTTTGGAAGACCTGTTAATGGGCACACTAGGAATTGTGTGCCCCATCTGTTCTCAGAAACCATAATCTACCATGGCTGATCCTGCAGCAGCAACGAAGTATCCTCTCCTGAAATTATTAGGCAGCACTTGGCCAACCACTCCGCCGCGACCCATACCAAAGCCGTCGCCTTGGGCACCGAAGAAACACAGACGACTATCCAGCGACCAAGATCAGAGCCAGACACCGGAAACCCCTGCCACACCACTAAGTTGTTGCACAGAGACTCAGTGGACAGTGCTCCAATCCTCACTGCATTTAACAGCTCACACAAAGGACGGATTAACTGTAATAGTAACACTACACCCATAGTACATTTAAAAGGTGATGCTAATACTTTAAAATGTTTAAATATAATTTAAAAAATTGTACATTGAAACCGCAG  CCCACAGGCTGAAAGAGAAA |
| CT 871 | 11q | CTGTTTCTGAAACCCTGTCCTTTGTGTGTCCGTGGTGTGCATCCCAGCAGTAAGCAACAATGGCTGATCC  AGAAGTGTTCATGAGCTTTGGAAAAGCTGCTAATAAGCCCTCAATCCGAGTTCGGGTCATTTCCACAAACTGTCGAGAAACAATAGCCTTTCCTGCTTTTGTGCAGACCGCTGCTGCCAACAGCACCTGCCAGGAACATATGCATAAGTACACTGGACGATGTTTTAAATCAAGTTATCTATTGACAAAAAAAAAAA |
| CT 896 | 13q | CTTCGGTTGTGCGTGCAAGCACACACGTATACATTCGTACTTTGGAAGACCTGTTAATGGGCACACTAGGAATTGTGTGCCCCATCTGTTCTCAGAAACCATAATCTACCATGGCTGATCCTGCAGGACC  CTTCTAGAGAACATTTGCCTTCGGCAACGGACTCTGAAGGTCACTGTCCTCTGGAGCTTAAGAACCAATATAATCATAGAATCCAGCCACTCAATAAATCCCAGCATCATACTCCAAAAGAGCTGCTGTTTCCAATTTCTTTATTCTTTGTAAAAAATATGGATAGGGATTTGTTTGATAACGAGCGCTGAAAAATTTTTTAAAAAAAAAAAAATTAAGAA |
| CT 976 | 13q | CTTCGTTGTGCGACAACCCCGTAACTTCGTACTTTGAAACCTGTTAATGGGCACACTAGGAATTGTGTGCCCCATCTGTTCTCAGAAACCATAATCTACCATGGCTGATCCTGCAGGT  TGCACAAAAGTTTATACCAAGTCTTCTCATTTAAAAGCTACCTGAGGACTACACTGGTA |
| CT 927 | 13q | CTTCGGTTGTGCGTACAAGCACACACGTAGATCTTCGTACTTTGGAAGACCTGTTAATGGGCACACTAGGAATTGTGTGCCCCATCTGTTCTCAGAAACCATAATCTACCATGGCTGATCCTGCAGGTGCATTAGCTGAAAAAAGCCACTGTGTCCTGGGATGGTTTTAGACATCTGGACAAAAGGCAAAGATTCCATAATATAAGGGGTCGGTGGACCGGTCGATGTATGTCTTGTTGCAGATCATCAAGAACACGTATAGAAACCCAGCTGTAATCATGCATGGAGATACACCTACATTGCATGAATATATGTTAGATTTGCAACCAGAGACATCTGATCTCTACTGTTATGAGCAATTAAATGACAGCTCAGAGGAGGAGGATGAAATAGATGGTCCAGCTGGACAAGCAGAACCGGACAGAGCCCATTACAAT  CTCCAGCATTCCCCCTCAAACCTAAAAAAAAAAAAAAAAAAAAAG |
| CT 1094 | 13q | CTTCGGTTGTGCGTGCAAGCACACACGTATACATTCGTACTTTGGAAGACCTGTTAATGGGCACACTAGGAATTGTGTGCCCCATCTGTTCTCAGAAACCATAATCTACCATGGCTGATCCTGCAGGACC  CTTCTAGAGAACATTTGCCTTCGGCAACGGACTCTGAAGGTCACTGTCCTCTGGAGCTTAAGAACCAATATAATCATAGAATCCAGCCACTCAATAAATCCCAGCATCATACTCCAAAAGAGCTGCTGTTTCCAATTTCTTTATTCTTTGTAAAAAATATGGATAGGGATTTGTTTGATAACGAGCGCTGAAAAATTTTTTAAAAAAAAAAAAATTAAGAA |
| CT 914 | 17q | GKGKGGGTMAGGCACACACGTAGACATTCGTACTTTGGAAGACCTGTTAATGGGCACACTAGGAATTGTGTGCCCCMTCTGTTCTCAGAAACCATAA TCTACCATGGCTGATCCTGCA  GCTGGAGTCTCACTGTCGCCCAGGCTGCAGTGAGTGAAGTGGTGCGATCTTGGCTCACTGCAACATCTGCCTCCCGGGTTCAAGTGATTCTCCTGCCTCAGCCTCCTGAGTAGCTGGGACTACAGGCACACACCGCAACGCCCGGATGATTTTTTGTATTTTTAGTAGAGATAGAGTTTCACCGTGTTGCCCAGGCTGGTGTCAAACTCCTGAGCTTAGGCGATCCACCCGCCTCAGCCTCCCAAAGTGCTGGGATTACTGGCTTGAGCCGGTGCGCCAGGCCCAAATTTATTTTACTTTCAAAGCCACATGTATAAAACTTAGTTATATAGTTTTAACAGCTTTATGTAATAATTTTATTGAAAACATTATTCTTTTATTGTTCAAAATGTTCTATATGTTACGTGCAATTTTGCTTTCCTTGTTGCTCTGTAGTTTTAAAAGTTCTTTGTTTTTCTAAAAACAACTTCAGTATATTTTTTAATTATTTGTATAATTTGGCGGCTCTTATTGTGATTTTAGAATAATATTGTTTATGAACTAAATATATATATTCTCATTTGCCTTTAAAAAAAAAATAAATAAAKRRSWSYRYRCYATSAACATRS |
| CT 848 | 17q | CCTTCGGTTGTGCGTACAAGCACACACGTAGACCG  TATTTCTCAATGTGAGAAGGACATGATATTTGGGAGGCCAGGGGTGGAATGATATGGTCTGGATGTGTGTCCCCTCCAAATCTCATGTTGAAATGTGATTCCCAGTGTTGGAGGTGGGGCTCAGTGGGAGGTAATGGATCATGGTGATGGGTGAGGAATTCATGCTTGAGTTAGTTCACGTGAGATCTGGCTGTTTAAAAGAGTCTAGGGCTAGGCATGGTGGCTCACACCTGTAATCCCAGCACTTTGGGAGGCCAAGGCAGGCAGTTCACTTGAGCGCAGGAGTTTGAGACCAGCCTGGGCAACATGGCAAAACAAAAA |
| CT 1097 | 18q | CTTCGGTTGTGCGTACAAGCACACACGTAGACCTTCGTACTTTGGAAGACCTGTTAATGGGCACACTAGGAATTGTGTGCCCCATCTGTTCTCAGAAACCATAATCTACCATGGCTGATCCTGCAGG  GGGAAGCTCATGCCACAGGACCTCTTCAAGAGCTGGAGACAAAAAGCCCCCTGTTCTGAGAATTGTTGAAAGGTTTCTTTCCCTTTCCCAGATTCTTTGCTCCTCGGTGCCCTGTCAAAACATCTGGCCCCTTTTGTCCTCAGCTTTCCCTGTGCTATTAAAGCCAGTCCTCATCACGAGCCACTTCCTGTGGACCTAAACATTTTTTTTATTATGCACCTTAACACTTCAAGATGATTAGAGCTGAAAGGGTAAGAACAAAAAATGAAGTTGCGCCTTTCTTTCACACTAATTTGATTC |
| CT 740 | 18q | CTGTTTCTAAACCCTGTCCTTTGTGTGTCCCTGGTGTGCATCCCAGCAGTAAGCAACAATGGCTGATCCAGAAGGT  TAATGTTTGATCAAGATCTTCAGCTCGAAGGAGGAGAAGGAAGGGATCGGGTTTGCATGGATATGAGTGACCCACTTCACATGGAAA |
| CT 906 | 19p | CTTCGGTTGTGCGTACAAAGCACACACGTAGACATTCGTACTTTGGAAGACCTGTTAATGGGCACACTAGGAATTGTGTGCCCCATCTGTTCTCAGAAACCATAATCTACCATGGCTGATCCTGCAGCTAC  TCAGGAGTCTGAGGTGGGAGAATTGCTTGAACCCGGAAGGCAGAGACTGCTGTGAGCTGAGATTGTACCACTGTACTCTACCTGGGCCACAGACCAAGACTCTGACAAAAAAGAAAGAAAGAAAGAAAGTAAAGAATGAAAAAAAGAAAGAAGAGAA |
| CT 889 | 19q | CTTCGGTTGTGCGTACAAGCACACACGTATCACATTCGTACTTTGGAAGACCTGTTAATGGGCACACTAGGAATTGTGTGCCCCATCTGTTCTCAGAAACCATAATCTACCATGGCTGATCCTGC  AGGTAGCTGAAGCAGAGAGAGAAGGCAGCATACATCAGCAATTTCTTCTCTGCACTTATAAGATCAAAGACTTTAAGACTTTCACTATTTCTTCTACCACTATCTACTACAAACTTCAAAGAGGAACCAGGAGTGTGAGAGGAGCATGAAAGTGGACCAGGAACGTGACCATTGAAACACTCCACCACAGGGAGGGGTTTCCGCCTCGAGATGACTGCCTCCAGGCCTAGATAGAATCCAGGCTTCCCAAGAATCCGGCGGAACAAATGTACCCCACTCCCCCAAGGAAC/ATGGAGACGTCCCTTT/ATGTGGTCTGCTAAGTAACGGTGCTGTCCCAAACACTAACGGTTAACCACTATGACCAAGGAGCCCTCCAAGCAGCTCCTTACTGTGAGAGTGTAACTAGAGGTCTCTCCTTGCTTCTAGGTGCCTACTATTCTGTCCACCACTGGTCCCCTTGCTAGCGACAACACGGTCTTAATTTCCCTTACAGGCTCATTATTACTAATTTAGCCGCCAACAACACACACACGAAACAGACGATTCCCCTCTAGAT |
| CT 839 | 20q | CTTTCGGGTTGCGCGTACAAGCACACACGTACAATTCGTAATATAGGAAGACCTGTTAATGGGCACACTAGGAATTGTGTGCCCCATCTGTTCTCAGAAACCATAATCTACCATGGCTGATCCTGCAGC  TGGCTGGATGTTGATCCTCAGGGAGACCTTGGAAGCTACCTGTTGCAGATGACTGAGCCCCCATCTGTCAGATTCCTGAGTGACTGACTGCATGAAGCAGAGCTCCTCCTCCTCATTGACCATCCTAACAATAAGAAATGTCTGCATTTACAAATGAAATGTTGTAATACTATGAAATTTTAAGAATTGAAATAAAAAATACAACTTATAAAAAAAAAAAAAAAAAAAAAAAAATCGATGTCCACTCCAGTCATCTTGCTGAAAAACTCCA  GCCATCCGAAAGATCTGGCGGCCGCTCTCCCTATAGTGAGTCTTATTACGCCGGATGGATATGGTGTTCAGGCACAAGTGTTAAAGCAATTGATTTTATTCACTATGATGAAAAAAACAATGAATGGAGCCTGCTCCAAGTTAAAAATAGAAATAATACCGAAAACTCATCGAGTAGATTAATTAGAGATAATACAACAATAAAAGAATGGTTTATAACTTACTCACAGCGTGATGCTACTAGTTGGGACATTTTCCAGATGATGTATCATCTATTAATTTGAATGAAAAAGACTACCTGCTTTTGTTAAAATTATTTGACAAAAATAATATCATTCGGCTGCAGGGCGGCCTCGTGATACGCCTATTTTTATAGGTTAATGTCATGATAATGATGGCTTCTAGACGTCAGGTGCTATTTTTCGGGGAATGTCGCAGCACCCCTATTTATTTTTTTTCTAATACATCAAGTATGTATCCG  CTCATGAAACACTAGCTGATGATGCTCAATAGATGAAAAAGAAAAAGATGGATATCACTTCGGGTCCCTTCTTCTTTTTGCGATTGCCTCTGTCTGCCCACAAACGGAGAATAAGAGCTAACGTGGGCAAGGGTTACAAGTACCAGGAACTGGATCCAATCTTAAGAAACTTACGT |
| CT 785 | 20q | CCTTCGGTTGTGCGTACAAGCACACACGTCGACATCCGTACGTTGGAAGACCTGTTAATGGGCACACTAGGAATTGTGTGCCCCATCTGTTCTCAGAAACCATAATCTACCATGGCTGATCCTGCAG  TATTTCCTCCAAAGCCGATCACCTTGACGGACGAGCGGAAAGCCCACCGGCTGCAGCGCATGCTGGACATGAAGGTGAATCCTGTGCAGGGCCTGGCCTCCCGCTGGGACTATGAGAAGAAGCATTGGAACATGTGGTTTGCATCACCTTCTGACTCCCTGAGGCTCCGCCCTGGCTGGGAGCCTCTGGCGGC |
| CT 1170 | 20q | CGGTTGTGCGACAAGACCACGTACACATCCGTACGTTGGAAGACCTGTTAATGGGCACACTAGGAATTGTGTGCCCCATCTGTTCTCAAAACCATAATCTACCATGGCTGATCCTGCA  TATTTCCTCCAAACCGATCACCTTGACGGACACGGACCTGGCA |
| CT 918 | 20q | CCTTCGGTTGTGCGTACAAAGCACACACGTACTACATCCGTACGTTGGAAGACCTGTTAATGGGCACACTAGGAATTGTGTGCCCCATCTGTTCTCAGAAACCATAATCTACCATGGCTGATCCTGCAG  TATTTCCTCCAAAGCCGATCACCTTGACGGACGAGCGGAAAGCCCAGCAGCTGCAGCGCATGCTGGACATGAAGGTGAATCCTGTGCAGGGCCTGGCCTCCCGCTGGGACTATGAGAAGAAGCAGTGGAACAATGACTTGCATCCCCACTGTCTCCCTGAGCTCCCCCTGGTGGACCTCTGGCCCTCCCCTCCCCTGCCCTAACCCCAAATCAA |

Key: HPV sequences are highlighted in blue.

Table S3b. Sequences of site of viral integration with low score

| **Sample ID** | **Sequence** |
| --- | --- |
| CT 1123 | CCTTTCGGTTGTGCGTACAAGCACACACGTACTACATTCGTACTTTGGAAGACCTGTTAATGGGCACACTAGGAATTGTGTGCCCCATCTGTTCTCAGAAACCATAATCTACCATGGCTGATCCTGCAGATCATCAAGAACACGTAGAGAAACCCAGCTGTAATCATGCATGGAGATACACCTACATTGCATGAATATATGTTAGATTTGCAACCAGAGACAACTGATCTCTACTGTTATGAGCAATTAAATGACAGCTCAGAGGAGGAGGATGAAATAGATGGTCCAGCTGGACAAGCAGAATCGGACAGAGCCCATTACAAT  CTCCAGCATTCCCCCTCAAACCTAAAAAAAAAAAAAAAAAA |
| CT 1194 | CTTCGGGTTGTGCGTACAAGCACACACGATCAATTCGTACATAATGGAAGACCTGTTAATGGGCACACTAGGAATTGTGTGCCCCATCTGTTCTCAGAAACCATAATCTACCATGGCTGATCCTGCAGGAGCAACAAAGTATCCTCTCCTGAAATTATTAGGCAGCACTTGGCCAACCACTCCGCCGCGACCCATACCAAAGCCGTCGCCTTGGGCACCGAAGAAACACAGACGACTATCCAGCGACCAAGATCAAAGCCGGACACCGGAAACCCCTGCCACACCACTAAGTTGTTGCCGGAGACTCGTGGACAATGCTCCAATCCTCACTGGATTTAACAGCTC  CCGGAGGATTAAAAAAGGAGTAACCCCACACCCAAAAAAATT |
| CT 777 | TTTGCWKGGGAKSGAAGGSCCGGGGGCGRAAGYAACACGTARACATTCGTACTTTGGAAGACCTGTTAATGGGCACACTAGGAATTGTGTGCCCCMTCTGTTCTCAGAAACCATAATCTACCATGGCTGATCCTGCAGSAGCAWCRAAKKATCCTCTCCTGAAATTATTAGGCAGCACTTGGCCAACCACTCCGCCGCGACCCATACCAAAGCCGTCGCCTTGGGCACCGAAGAAACACAGACGACTATCCRGCGACCAAGATCARAGCCAGACACCGGAAACCCCTGCCACACCACTAAGTTGTTGCACAGAKACTCRGTGGACAGTGCTCCAATCCTCACTGCATTTAACAKCTCACACRAAGGACGGATTAACTGTAATAGTAACACTACACCCATAGTACATTTARRAGGTGATGCTAATACTTTAAAATGTTTAAGATATAGATTTAAAAAGCATTGTACATTGTATACTGCAGTGTCGTCTACATGGCATTGGACAGGACATAATGTAAAACATAAAAGTGCAATTGTTACACTTACATATGATAGTGAATGGCAACGTGACCAATTTTTGTCTCAAGTTAAAATACCAAAAACTA  AAAAAAAAAAAAAAAAAAAMTAATGTCCTCCGGACCCCCAATYACCCTTGTCGRCCT |
| CT 733 | CTTTCAGTTGTGCGTACAAGCACACACGTACACATTCGTACTTTGGAAGACCTGTTAATGGGCACACTAGGAATTGTGTGCCCCATCTGTTCTCAGAAACCATAATCTACCATGGCTGATCCTGCAGCAGCAACGAAGTATCCTCTCCTGAAATTATTAGGCAGCACTTGGCCAACCACTCCGCCGCGACCCATACCAAAGCCGTCGCCTTGGGCACCGAAGAAACACAGACGACTATCCAGCGACCAAGATCAGAGCCAGACACCGGAAACCCCTGCCACACCACTAAGTTGTGCACAAACTCGACAGTGCTCCTCCTCACTGATTTAACAGCTCACACAAAGGACGGATTAACGCTGTAATAGTATACACTACACCCCATGTACATTTAAAAGGACGATGCATAAACCTTAAAATTGCTTTAACCATAAAAATTTATAAAA  CACCATCAAATCTTCGAAATAACCTCGACAGTGTCCCTCCAAAAGTTTGCCTTCTAACTACAAACAAAACACATTAGACTCACACTCCATACATTAACATATTACCGATTCAAAATATAAAGAACAAATACATAAAAAAAATAATAGTAGATATGATCTAAATTTCTACC |
| CT 718 | CTTTCGGTTGTGCGTACAAGCACACACGTAGCCATTCGTACTTTGGAAGACCTGTTAATGGGCACACTAGGAATTGTGTGCCCCATCTGTTCTCAGAAACCATAATCTACCATGGCTGATCCTGCAGGTGTATTAACTGTCAAAAGCCACTGTGTCCTGAAGAAAAGCAAAGACATCTGGACAAAAAGAAAAGATTCCATAATATAATGGGTCGGTGGACCGGTCAATGAATGTCTTGTGGCCGATCCCGGCAACACTGTTGGAACCCCAGGTGTAACCCTGCCTGGTGATACCCCTACATTCATTGCTGGTATGTTCAATTTGCCCCCCCAGACTACTGATCTCTACTGTTATGACCAATTACATGACAGCTCACTCTAGGAGGATGAAATAAATGGTCCAGCTGGACAAGCAGAATCGGACAGAGCCCATTACAAT  CTCCAGCATTCCCCCTCAAACCTACCACACCCCCTAAAAAAAAAAAAAAAAAAAAAAAAAAAAAAAAAAAAAAACAAAGAACATGTTATTTATTCCTCCCGCAGACCCCCGAAAAACAACCATTTTAAACAACCCCACATAGAAAAGGAAAA |
| CT 707 | CTTCGGTTGTGCGTACAAGCACACACGTATACATCCGTACGTTGGAAGACCTGTTAATGGGCACACTAGGAATTGTGTGCCCTATCTGTTCTCAGAAACCATAATCTACCATGGCTGATCCTGCAGCAGCGACGAAGTATCCTCTGCTGAAATTACTAGGCAGCACTTGGCCAACCACTCCGCCGCGACCCATACCAAAGCCGTCGCCTTGGGCACCAAAGAAACACAGACGACTATCCAGCGACCAAGATCAGAGCCAGACACCGGAAACCCCTGCCACACCAACAAGTTGTTGCACAGAGACTCCGTGGACAGTGCTCCAATCCTCACTGCAGTTAACAGCTCACACAAAGGACGGATTAACTGTAATAGTAACACTACACCCATAGTACATTTAAAAGGTGATGCTAATACTTTAAAATGTTTAAGATACAGATTTAAAAAGCATTGTAAATTGTATACAGCAGTGTCGTCCACATGGCATTGGACAGGACATAATGTAAAACATAAAAGTGCAATTGTTACACTTACATATGATAGTGAATGTCAACGTGAACAATTTTTGTCTCAAGTGAAAATACCAAAAACTAATACAG  AAAAAAAAAATATATATTAGCTA |
| CT 819 | CCTTTCGGTTGTGCGTAAAAGCACACACGTATACATTCGTACTTTGGAAGACCTGTTAATGGGCACACTAGGAATTGTGTGCCCCATCTGTTCTCAGAAACCATAATCTACCATGGCTGATCCTGCAGGT  GTATTAGCTGTCAAAAGCCACTGTGTCCTGAAAAAAAGCCAATACATCTGCTGGATTCACACCAATCCCCTGCCACACCTCCGGATGGATGGAACTATGTATGCCCGCCTGCACACCCGGTAATTTTTTTATAATCCCATATGAAACTGTGTGACCGATACCCGAGGGTGCATCTCCTGAACTCGAAACAAACACGATCTCCAAATGAAAGGCCCCAAACAACCA |
| CT 959 | GTKTKCSGTMAGCACACACGTAGACATTCGTACTTTGGAAGACCTGTTAATGGGCACACT  AGGAATTGTGTGCCCCAYCTGTTCTCAGAAACCATAATCTACCATGGCTGATCCTGCAGS  AGCAACRAAKKATCCTCTCCTGAAATTATTARGCAGCACTTGGCCAACCACTCCGCCGCG  ACCCATACCAAAGCCGTCGCCTTGGGCACCGAAGAAACACAGACGACTATCCAGCGACCA  AGATCARAGCCAGACACCGGAAACCCCTGCCACACCACTAAGTTGTTGCACAGAKACTCA  GTGGACAGTGCTCCAATCCTCACTGCATTTAACAGCTCACACAAAGGACGGATTAACTGT  AATAGTAACACTACACCCATAGTACATTTAAAAGGTGATGCTAATACTTTAAAATGTTTA  AGATATAGATTTAAAAAGCRTTGTACATTGTATACTGCAGTGTCGTCTACATGGCATTGG  ACAGGACATAATGTAAAACATAAAAGTGCARTTGWGACACTTACATATGATAGTGAATGG  CAACGTGACCAATTTTTGTCTCAAGTTAAAATACCAAAAACTA  ATAWAAAAAAAAAAAAACAAC |
| CT 912 | CTTCGTTGCGCGTACAAGCACACACGTCACATTCGTACTAATTGGAAGACCTGTTAATGCGGCACACTAGGAATTGTGTGCCCCATCTGTTCTCAGAAACCATAATCTACCATGGCTGATCCTGCAGCACAACGAAGTATCCTCTCCTGAAATTATTAGCAGCACTTGGCCAACCACCCCGCCGCGACCCATACCAAAGCCGTCCCTTGGGCACCGAAAAACACAACACTATCCACGACCAA  ATACAACCCAACCCCTGACATATTGGAAACAAGAAG |
| CT 837 | CTTCGGTTGTGCGTACAAGCACACACGTAGTCCATTCGTACTTTGGAAGACCTGTTAATGGGCACACTAGGAATTGTGTGCCCCATCTGTTCTCAGAAACCATAATCTACCATGGCTGATCCTGCAGCAGCAACGAAGTATCCTCTCCTGAAATTATTAGGCAGCACTTGGCCAACCACTCCGCCGCGACCCATACCAAAGCCGTCGCCTTGGGCACCGAAGAAACACAGACGACTATCCAGCGACCAAGATCAGAGCCAGACACCGGAAACCCCTGCCACACCACTAAGTTGTTGCACAGAGACTCAGTGGACAGTGCTCCAATCCTCACTGCATTTAACAGCTCACACAAAGGACGGATTAACTGTAATAGTAACACTACACCCATAGTACATTTAAAAGG  AGGTAGGAGGAGGTGAGAAAATTACTACTTGATGTCCTCCTCCTGCCGTATCACCGCTGGTGGTATATTCTCATCGGAGGGCGGCGACCATTGTGGACAGTGAATGAGGGGAATCTTCGATGCTTGATGCTTATTATGATCCGACGAGGAGTGCTGGATAATCTTCGAGGAGCCACCTCTTCTCAAAAAACCCCCCCCACCCTCCTTCCTTGGTGAAAAATTTGGGGAGGCCCCCCTGGGGGAGACAAGAGGGGGATGCTGCCCAAAAGGCGAAAAAAGGAAAAAAACTTCCTCCTTGTTTTTCTATCACCCACGTCCAAAAAATTAGCTCTGTCGTGTACGTGTGTGTACGTGTGCCACAACCCACTACACTATATAGAAAGAGATGAATGTACAATATACG |
| CT 744 | GTGGAAAGGCTTCGGTTGTGCGTAAGGCACACACGTAATCCTTCGTACTTTGGAAGACCTGTTAATGGGCACACTAGGAATTGTGTGCCCCATCTGTTCTCAGAAACCATAATCTACCATGGCTGATCCTGCAGGTACAAATGGGGAAGAGGGACGGGATGTAATGGATGGTTTTATGTGCAGGCTGTAATGAAAAAA  TTCTCCTGCCTCAATGGAGTATGGCTCCAGCCAACAAACAGATAGTTGGAAAA |
| CT 763 | CTTCGGTTGTGCGTACAAAGCAACACGTATAATTCGTGCTTTGGAAGACCTGTTAATGGGCACACTAGGAATTGTGTGCCCCATCTGTTCTCAGAAACCATAATCTACCATGGCTGATCCTGCAGGTACCAATGGGGAAGAGGGTACGGGATGTAATGGATGGTTTTATGTAGAGGCTGTAGTGGAAAAAAAAA  AAAAAAAATCAATGTCACTCGGGTCAAAAAATCGGAGAGCCCCTTAAAATCCCCCGCATTCCCCCCCCAACCTAAAAAAAAAAAAAAAAAAAAAAAAAAAAAAAAAAAAAAAAAAA |
| CT 793 | CTTCGGTTGTGCGTACAAGCACACACGTAGACATTCGTACTTTGGAAGACCTGTTAATGGGCACACTAGGAATTGTGTGCCCCATCTGTTCTCAGAAACCATAATCTACCATGGCTGATCCTGCAGGTACCAATGGGGAAGAGGGTACGGGATGTAATGGATGGTTTTATGTAGAGGCTGTAGTGGAAAAAAAAA  AAAAAAAAACGGGGTCAATCCAGGCAATCTAAATGGCTTTCGAGGACACAAGACCCAAATCCGGGGTGTTTTTTTTACAACGCCCGAACGGGAAAAACTGGTGGTCCCCTTAATCCCCTGCAGCA  AATCCCCCTTTATAATTGGGGGG |
| CT 1215 | CTTCGTTGTGCGTACAAGCACACACGTAGACATTCGTACTTTGGAAGACCTGTTATGGGCACACTAGGAATTGTGTGCCCCATCTGTTCTCAGAAACCATAATCTACCATGGCTGATCCTGCAGGTACCAATGGGGAAGAGGGTACGGGATGTAATGGATGGTTTTATGTAGAGGCTGTAGTGGAAAAAAAAA  AAAAAAAATCGATGTCACCCCAGTCAAAAAAAATGCGTTTTCGAGGG  GCCCAGATCAATTTTCTGGGCGGCGTTTTTTAACGGCGTGGCTGGGAAAAACCGGGTTTCCCCACTTTTCCCCTTGGGCCATCCCCCCTTAAAGTTGGAGGGGGCCCTGGGTTAAGGGCCGGGTCTGATTTGAAAGACC |
| CT 825 | CCTTCGGTTGTGCGTACAACGCACACACGTAGACATTTCCTACTTTGGAAGACCTGTTAA  TGGGCACACTAGAGAATTGTGTGCCCCATCTGTTCTCAGAAACCATAATCTACCATGGCT  GATCCTGCAGCAGCAACAAAGTATCCTCTCCTGAAATTATTAGGCAGCACTTGGCCAACC  ACTCCGCCGCGACCCATACCAAAGCCGTCGCCTTGGGCACCGAAGAAACACAGACGACTA  TCCAGCGACCAAGATCAGAGCCAGACACCGGAAACCCCTGCCACACCACTAAGTTGTTGC  ACAGAGACTCAGTGGACAGTGCTCCAATCCTCACTGCATTTAACAGCTCACACAAAGGAC  GGATTAACTGTAATAGTAACACTACACCCATAGTACATTTAAAAGGTGATGCTAATACTT  TAAAATGTTTAAATATAATTTAAAAAGCATTGTACATTGTATACTGCAGTGTCGCTACTG  GATTGGACAGCGGATGTAAACATAAAAAGTGCAACTTGTCTACCCTTCAT  CATACGAGGTGGAACGCTCACTTTGTCCACAATTAACATCATACTAAAAAAAAA |
| CT 752 | CGTTTCTGAAACCCTGTCCTTTGTGTGTCCTCTGGTGTGCATCCCAGCAGTAAGCAACAATGGCTGATCCAGAAGGTACCTACGGGGACGGAACGGGTTGAAACGGCTGGTTTTATGTACAAGCTATTGTAGACAAAAAAACTGGAGATGTAATATCGGATGACGAGGAGGAAAATGCGGTTCGGGCTGGGTTGCTACACGACGGGAAGTTGCCACACACGGACATTTTGTGAGCAGGAAAATATAGAGGCTGACACGAATTGTTCCCTGCCAAGAGACCACAATGATGCACACTGTTGCATGTTTTAA  CACCAACCTTCCCCCACCCACAACACCCCCCCCCAAAAA |
| CT 1202 | CCTTCGGTTGTGCGTACAAAAGCACACACGTTCGACATTCGTACTTTGGAAGACCTGTTAATGGGCACACTAGGAATTGTGTGCCCCATCTGTTCTCAGAAACCATAATCTACCATGGCTGATCCTGCAGGT  GCATTAGCTGTCAAAAGCCCCTGTGTCCTGAAATGGTTTTATGCATAGGCTGAAATGGAAAAAAATCCCTGGTATAATGGGTCGGTGGACCGGACGATGTTTGTCTTGTTGCAGATCATCAAGAATTCGTACAGAAACCCAGCTGTAATCATGTGCAGATCCAACATAGCTGATTTGCCCATTCCCCCCCGACACACCGACCCACTGCAAACA |
| CT 716 | CGCTCGCGACTTACAAACAGATCCATAAACCCCGTCCTTCGGTTGTGCGTAACAAAGCACACACGCTAGTACATTCGTACTTTGGAAGACCTGTTAATGGGCACACTAGGAATTGTGTGCCCCATCTGTTCTCAGAAACCATAATCTACCATGGCTGATCCTGCAG  AAATTATTCTCCTCCTGGTGGTGCTGGCAGAACAACCCCTGGTGGCCCCACTCTTCCTCCAATACTTATTTCATCCTCCTGAGGAGGACGCCCTGCTGCTCCCCCGCTGGCTGCTTTTTTTCAGGAAGTGGTGGCCAAAAATAATCATCCTCCACCCCCCCCCAACAGTTATGTTTGTTTCTTCTCTTGGGGGAAAAAATAAAAGTGGTGTTTATTAAAATTTTTTTTTGAAGAAAAAAAAGATAAAAAAAGGACTTGGTGATGATTTTTTTTTTGTTAAAAAAAACCCCCCAACAATTTTTTTTTTAACAAAAAAAAAACCTGAAGGGGGGGGGGGGGAAGAATTTATAATACAATGCTGCCCGCTATAAAAGATGCACCACACATGATGACTACTGCCGTCTTCTTGTCGCCGCGTGGGGCGAGAGTCGTGGCGACCACCACAAAATAATACGTGGTGATCACATCATGGTGATGAGTATTAGCAGCATATAGAGCAAAGGTGGGGAAATTCTTCACGATGTTCATCAGAAGTGAAGAAACATAAAAAACACTTCTTCCACCACCTCATATAACCACCACTTTTTCTGGCGCACACCGGGTTGCTCCTCGTCCTACTACCCCTTCTTCTGTCGGCTGTGCTCCGGAGCTAGCTGTCTTCTTCGCTGCCTCCTTGCTGACTAATAACTCCCCCTTCTAAAAAAAAAAAAAAAAAAATTTGTTCACCTAACCCCACACAACTCAGAAGACCCCTTTTTTCCTTTATTCCCCCCCCACTTTAAAAAAAAAAAAGAAAGGGGAAAC |
| CT 940 | CTTCGGGTTGTGCGTACAAGCACACACGTCGACATTCGTACTTTGGAAGACCTGTTAATGGGCACACTAGGAATTGTGTGCCCCATCTGTTCTCAGAAACCATAATCTACCATGGCTGATCCTGCAGGGCCAACGAGATATCCTCTCCTGAAATTATTAATGCAGCACTTGGCCAACCACCCCGCCGCGACCCATACCAAAGCCGTCGCCTTGGGCACCGAAGAAACACAGACGACTATCCAGCGACCAATATCAGAGCCAGACACCGGAAACCCCTGCCACACCACTAAGTTGTTGCACAGAGACTCAGTGGACAGTGCTCCAATCCTCACTGCATTTAACAGCTCACACAAAGGACGGATTAACTGTAATAGTAACACTACACCCATAGTACATTTAAAAGGTGATGCTAATACTTTAAAATGTTTAAGATATAAATTTAAAAACATTGTAATTGTATACTGCATTGTCGTCTACTGGATTGGACGAAATAATGT  TCAACTCACTCTTCCCCCACCACAG |
| CT 922 | AGGGTKGKCGGWAAAGCAMACACGTAGACATTCGTACTTTGGAAGACCTGTTAATGGGCACACTAGGAATTGTGTGCCCCMTCTGTTCTCAGAAACCATAATCTACCATGGCTGATCCTGCAGGTGTATTAACTGTCAAAAGCCACTGTGTCCTGAAGAAAAGCAAAGACATCTGGACAAAAAGCAAAGATTCCATAATATAAGGGGTCGGTGGACCGGTCGATGTATGTCTTGTTGCAGATCATCAAGAACACGTAGAGAAACCCAGCTGTAATCATGCATGGAGATACACCTACATTGCATGAATATATGTTAGATTTGCAACCAGAGACAACTGATCTCTACTGTTATGAGCAATTACATGACAGCTCAGAGGAGGAGGATGAAATAGATGGTCCAGCTGGACAAGCAGAAYCGGACAGAGCCCATTACAAT  CTCCAGCATTCCCCCTCAAACCTAAAAAAAAAAAAAAAAAAARAARWYKAAWCYAGGSCAAAGG |

Key: HPV sequences are highlighted in blue.
